# Unconventional-Phase 1T′-Transition Metal Dichalcogenide Monolayers Grown on Amorphous Templates for Highly Efficient Hydrogen Evolution

**DOI:** 10.1021/jacs.5c19857

**Published:** 2026-02-10

**Authors:** Zijian Li, Hua Yang, Mingjun Sun, An Zhang, Yiyao Ge, Xinyue Long, Biao Huang, Li Zhai, Wei Zhai, Lujiang Li, Lixin Wang, Chao Wang, Yanping Xu, Yanming Cai, Peigen Liu, Bo Chen, Lin Gu, Panzhe Qiao, Qinghua Zhang, Feng Ding, Hua Zhang

**Affiliations:** † Department of Chemistry, 53025 City University of Hong Kong, Hong Kong 999077, China; ‡ The Analysis & Testing Center, 12633 Beihang University, Beijing 102206, China; § Institute of Technology for Carbon Neutrality, Shenzhen Institute of Advanced Technology, 12381 Chinese Academy of Sciences, Shenzhen 518055, China; ∥ State Key Laboratory for Advanced Metals and Materials, University of Science and Technology Beijing, Beijing 100083, China; ⊥ National Synchrotron Radiation Laboratory, University of Science and Technology of China, Hefei 230029, China; # State Key Laboratory of Flexible Electronics & Jiangsu Key Laboratory of Smart Biomaterials and Theranostic Technology, Institute of Advanced Materials, 12577 Nanjing University of Posts and Telecommunications, Nanjing 210023, China; ¶ Beijing National Center for Electron Microscopy and Laboratory of Advanced Materials, Department of Materials Science and Engineering, 12442 Tsinghua University, Beijing 100084, China; ∇ Shanghai Synchrotron Radiation Facility, Shanghai Advanced Research Institute, Chinese Academy of Sciences, Shanghai 201210, China; ○ Beijing National Laboratory for Condensed Matter Physics, Institute of Physics, Chinese Academy of Sciences, Beijing 100190, China; ⧫ Research Division of Advanced Materials, Suzhou Laboratory, Suzhou 215133, China; †† Hong Kong Institute for Clean Energy (HKICE), City University of Hong Kong, Kowloon, Hong Kong 999077, China; ‡‡ Hong Kong Branch of National Precious Metals Material Engineering Research Center (NPMM), City University of Hong Kong, Hong Kong 999077, China; §§ Shenzhen Research Institute, City University of Hong Kong, Shenzhen 518057, China

## Abstract

Hydrogen is a promising clean energy carrier to address global energy and environmental challenges. Although platinum (Pt)-based catalysts are the benchmark for the hydrogen evolution reaction (HER), their high cost and scarcity limit their widespread application. Two-dimensional transition metal dichalcogenides (TMDs), particularly with the unconventional 1T′ phase, have emerged as promising alternatives, yet synthesizing them with high phase purity and stability remains challenging. Here, by using amorphous phosphorus (P)-doped Pd nanoparticles (*a*-PdP NPs) as templates, we develop a facile and general wet-chemical method to synthesize high-phase-purity and stable 1T′-TMD monolayers (MLs), including MoS_2_, WS_2_, and MoWS_2_, to construct *a*-PdP@1T′-TMD core–shell NPs. Experimental and theoretical analyses reveal that the formation and stabilization of 1T′-MoS_2_ MLs are attributed to the strong Pd–S interaction, electron donation from oleylamine, and amorphous nature of the template. The resulting *a*-PdP@1T′-MoS_2_ catalyst exhibits superior HER performance, requiring an overpotential of only −182.3 mV to achieve 1,000 mA·cm^–2^ and maintaining high stability for over 500 h at 500 mA·cm^–2^, outperforming the commercial Pt/C and placing it among the best reported MoS_2_-based catalysts. Impressively, the synthesized *a*-PdP@1T′-MoS_2_ can also be used as an efficient and stable support to grow single-atomically dispersed Pt with further enhanced HER activity, indicating its promise as a versatile platform for the design and preparation of advanced electrocatalysts.

## Graphical abstract

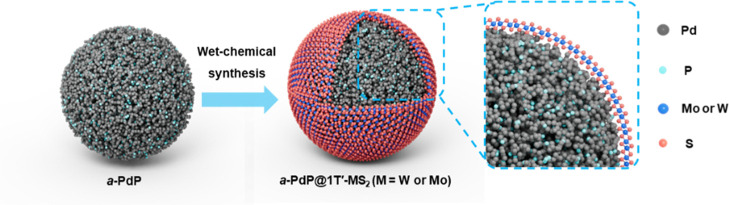

## 1 Introduction

Hydrogen (H_2_) is a promising clean energy carrier for addressing the global energy and environmental crises, owing to its high energy density and sustainability.^1,2^ Electrochemical water splitting, powered by efficient electrocatalysts, offers a viable path to green H_2_.^3,4^ To date, noble metal-based catalysts, particularly Pt, have been the benchmark materials for the hydrogen evolution reaction (HER) due to their superior activity.^5^ However, their widespread application is dramatically hampered due to the high cost and scarcity of noble metals. Therefore, developing high-performance, cost-effective HER electrocatalysts is of paramount importance.

Two-dimensional (2D) transition metal dichalcogenides (TMDs) have been used as promising electrocatalysts for HER, attracting extensive interest due to their large surface area and abundant active sites.^6,7^ Previous studies have shown that the edge sites of TMDs play a crucial role in optimizing the hydrogen adsorption free energy. For instance, MoS_2_, a typical TMD, exhibits an HER activity just below that of noble metals like Pt or Pd in the Sabatier volcano plot.^8^ Moreover, as layered 2D materials, TMD nanosheets normally display a thickness-dependent catalytic behavior, showing superior HER performance to their bulk forms.^9,10^

Importantly, TMDs are also polymorphic, featuring different crystal phases with distinct physicochemical properties.^11^ For instance, the group VIB TMDs (e.g., MoS_2_, WS_2_) primarily exist in the thermodynamically stable semiconducting 2H phase and also possess the metastable metallic 1T and semimetallic 1T′ phases.^12,13^ Recently, phase engineering of nanomaterials (PEN) has emerged as a powerful strategy to precisely regulate the atomic arrangement of TMDs for enhanced catalytic activity.^14–21^ For example, Liu et al. have reported that a few-layered 1T′-MoS_2_ flake grown by chemical vapor deposition (CVD) exhibits an overpotential of −205 mV to achieve 10 mA·cm^–2^, which is lower compared to the 2H-MoS_2_ (−286 mV) in HER.^20^ This improved performance is attributed to the higher basal-plane activity and superior electrical conductivity of the 1T′-MoS_2_.^19,20^ Recently, our group has prepared single-atomically dispersed Pt on 1T′-MoS_2_ nanosheets (*s*-Pt/1T′-MoS_2_), which delivers superior HER performance with an overpotential of −19 ± 5 mV to reach 10 mA cm^–2^, superior to that of HiSPEC 9100 Pt/C.^21^ Therefore, the synthesis of high-quality, atomically thin 1T′-TMDs is important for advancing electrocatalytic hydrogen evolution.

Crystalline metal substrates/templates have been used for synthesizing high-quality 2D TMDs.^22^ To date, synthetic strategies, including chemical vapor deposition (CVD)^23–27^ and molecular beam epitaxy (MBE),^28^ have been developed to prepare atomically thin TMDs. However, the aforementioned 2D TMDs prepared on crystalline metal substrates/templates normally crystallize in the pure 2H phase,^23,25–27^ or mixed 2H and 1T′ phases.^24,28^ Moreover, the metastable 1T′ phase tends to gradually transform into the thermodynamically stable 2H phase, hindering the investigation of its intrinsic catalytic properties and phase-dependent HER performance.^29^ Recently, by using unconventional 4H-phase Au nanowires as templates, our group has reported a facile wet-chemical synthesis of four single-layer semimetallic 1T′-TMDs, e.g., MoS_2_, WS_2_, MoSe_2_, and WSe_2_, with high phase purity and stability.^18^

Besides crystalline metal substrates/templates, amorphous nanostructures, which possess long-range disordered atomic arrangements, can also serve as an ideal substrate/template for growing a secondary material.^30^ Their rich unsaturated coordination sites with a high surface energy could facilitate the nucleation and growth of secondary materials. More importantly, compared to their crystalline counterparts, amorphous nanostructures could exhibit superior catalytic performance in various reactions owing to their abundant unsaturated coordination sites and dangling bonds.^31–34^ Our group has dedicated extensive efforts to the design and synthesis of diverse amorphous nanomaterials, including Pd_3_P_2_S_8_,^35^ Pd,^36–38^ Rh,^39^ and their based alloys.^39–41^ For instance, through lithiation-induced amorphization of crystalline Pd_3_P_2_S_8_, our group has successfully prepared amorphous lithium-incorporated palladium phosphosulfide nanodots, which exhibited significantly enhanced HER performance compared to the crystalline Pd_3_P_2_S_8_.^35^ Moreover, we have transformed the face-centered cubic (*fcc*)-Pd into the amorphous Pd (denoted as *a*-Pd) through a ligand-exchange strategy.^37^ The resulting *a*-Pd exhibited enhanced HER activity compared to the *fcc*-Pd. In another study, He et al. fabricated a wafer-size amorphous PtSe_*x*_ film via a low-temperature amorphization strategy, which also exhibited superior HER activity compared to the crystalline PtSe_2_.^42^ These findings highlight the catalytic advantages of amorphous nanostructures. Therefore, constructing hybrid nanomaterials by growing 1T′-TMDs on amorphous templates is anticipated to integrate the intrinsic catalytic merits of both components, thereby achieving enhanced HER performance, including high activity and long-term stability.

In this work, by using amorphous phosphorus-doped Pd nanoparticles (*a*-PdP NPs) as templates, high-phase-purity and stable 1T′-TMD monolayers (MLs), including MoS_2_, WS_2_, and MoWS_2_, have been synthesized to construct *a*-PdP@1T′-TMD core–shell NPs via a facile and rapid wet-chemical synthetic method (Figure 1a). Systematic characterization and theoretical calculations reveal that the formation and stabilization of high-phase-purity 1T′-MoS_2_ MLs on the *a*-PdP NPs can be attributed to their strong Pd–S interaction, charge doping from the oleylamine solution, and the unique amorphous nature of the PdP template. As a proof-of-concept application, the as-synthesized *a*-PdP@1T′-MoS_2_ NPs exhibit superior HER performance, requiring a low overpotential of −182.3 mV to reach 1,000 mA·cm^–2^ and featuring a low Tafel slope of 27.1 mV dec^–1^, thereby outperforming the commercial Pt/C and placing it among the best reported MoS_2_-based catalysts. In addition, the *a*-PdP@1T′-MoS_2_ catalyst also delivers ultrahigh stability, with negligible activity loss over 500 h at 500 mA cm^–2^. Moreover, it can serve as an ideal template for anchoring single-atomically dispersed Pt (*s*-Pt), thus, further boosting the HER performance.

**Figure 1:**
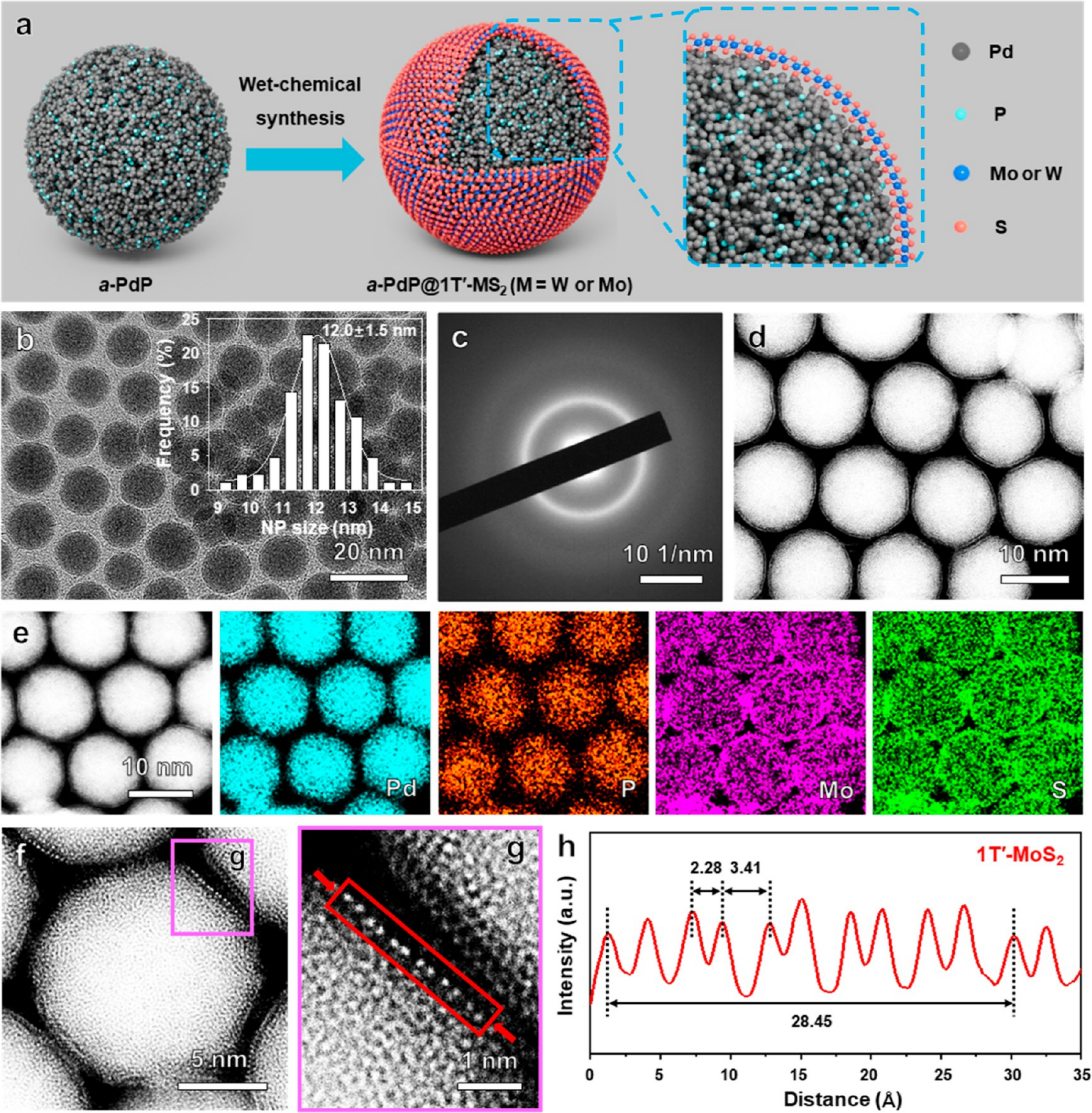
Synthesis and characterization of *a*-PdP@1T′-MoS_2_ NPs. (a) Schematic illustration of the wet-chemical synthesis of 1T′-TMD ML on *a*-PdP NP. (b) Low-magnification TEM image of the as-prepared *a*-PdP@1T′-MoS_2_ NPs. Inset: the corresponding size distribution histogram of (b). (c,d) SAED pattern (c) and HAADF-STEM image (d) of the as-prepared *a*-PdP@1T′-MoS_2_ NPs. (e) STEM image and the corresponding EDS elemental maps of the as-prepared *a*-PdP@1T′-MoS_2_ NPs. (f) Atomic-resolution HAADF-STEM image of a representative *a*-PdP@1T′-MoS_2_ NP and (g) the high-magnification HAADF-STEM image in the pink area in (f) showing the interface between 1T′-MoS_2_ ML and *a*-PdP NP. (h) The corresponding integrated pixel intensity profile of 1T′-MoS_2_ ML in the red rectangle in (g).

## 2 Results and Discussion

### 2.1 Synthesis and Characterization of *a*-PdP@1T′-MoS_2_ NPs

The *a*-PdP NPs with an average size of 11.3 ± 0.8 nm (Figure S1) were first synthesized based on a previously reported method^43^ with slight modifications (see Supporting Information for details). The energy-dispersive X-ray spectroscopy (EDS) analysis determined the Pd/P atomic ratio in the *a*-PdP NPs to be approximately 81.8:18.2 (Figure S2). By using the synthesized *a*-PdP NPs (Figure S1) as templates, 1T′-MoS_2_ MLs were grown on the *a*-PdP NPs to form the *a*-PdP@1T′-MoS_2_ core–shell NPs via a facile and rapid wet-chemical route (see Supporting Information for details). The synthesis of *a*-PdP@1T′-MoS_2_ NPs could be scaled up to ∼160 mg in a single batch of experiment (Figure S3). As shown in the low-magnification transmission electron microscopy (TEM) image (Figure 1b), the as-prepared *a*-PdP@1T′-MoS_2_ maintains the spherical morphology with a size distribution of 12.0 ± 1.5 nm, which is slightly larger than that of the *a*-PdP NPs (11.3 ± 0.8 nm, Figure S1) due to the grown 1T′-MoS_2_ MLs. The selected-area electron diffraction (SAED) pattern (Figure 1c) of the *a*-PdP@1T′-MoS_2_ NPs confirms the PdP NPs still maintain the amorphous phase after the growth of MoS_2_ MLs, which is further confirmed by the X-ray diffraction (XRD) pattern (Figure S4), suggesting a good structural stability of *a*-PdP. The high-angle annular dark-field scanning transmission electron microscopy (HAADF-STEM) image (Figure 1d) reveals that a MoS_2_ ML is uniformly grown on the *a*-PdP NP. The STEM-EDS elemental maps (Figure 1e) and STEM-EDS line scanning profiles (Figure S5) corroborate the even distribution of Pd, P, Mo, and S in the synthesized *a*-PdP@1T′-MoS_2_ NPs. Based on the STEM-EDS result, the Pd/P/Mo/S atomic ratio in the obtained *a*-PdP@1T′-MoS_2_ NPs is determined to be 59.6/9.3/9.2/21.8, which is in accordance with the corresponding inductively coupled plasma optical emission spectrometry (ICP-OES) result (Figure S6). The detailed crystal structure of 1T′-MoS_2_ was further investigated by atomic-resolution HAADF-STEM (Figure 1f,g), showing that the MoS_2_ ML grown on *a*-PdP NP possesses a pure 1T′ phase. The corresponding intensity profiles of MoS_2_ display two different alternating Mo–Mo projection distances of ∼2.28 and 3.41 Å (Figure 1h), which are in good accordance with the short and long Mo–Mo projection distances of 2.28 Å and 3.38 Å, respectively, in the crystal model of 1T′-MoS_2_ ML observed from the side view.^18^

Furthermore, Raman spectroscopy, X-ray photoelectron spectroscopy (XPS), X-ray absorption near edge structure (XANES), and extended X-ray absorption fine structure (EXAFS) were used to characterize the as-synthesized 1T′-MoS_2_ MLs with high phase purity on *a*-PdP NPs. For comparison, commercial 2H-MoS_2_ was also characterized. As shown in the Raman spectrum of the as-prepared *a*-PdP@1T′-MoS_2_ NPs (red curve in Figure S7), five distinctive peaks located at 150, 225, 250, 311, and 404 cm^–1^ (A_1g_), respectively, are observed, which are similar to the reported single-layer 1T′-MoS_2_.^20^ The characteristic Raman peak of 2H-MoS_2_ located at 380 cm^–1^ (E_2g_^1^) (blue curve in Figure S7) has not been observed in the *a*-PdP@1T′-MoS_2_ NPs, indicating the high phase purity of the synthesized 1T′-MoS_2_ MLs. XPS spectrum of *a*-PdP@1T′-MoS_2_ NPs (red curve in Figure 2a) shows two characteristic peaks located at 231.5 and 228.3 eV which can be assigned to the Mo 3d_3/2_ and Mo 3d_5/2_ of 1T′-MoS_2_, respectively.^20^ Both of them shift to a lower binding energy by ∼0.9 eV in comparison with Mo 3d_3/2_ (232.4 eV) and Mo 3d_5/2_ (229.2 eV) of 2H-MoS_2_ (blue curve in Figure 2a), which is consistent with the previous reports.^19,20^ Moreover, XANES and EXAFS measurements were conducted to investigate the electronic structure and coordination environment of the as-synthesized 1T′-MoS_2_. The absorption edge position of 1T′-MoS_2_ MLs on *a*-PdP NPs in the Mo K-edge XANES spectrum (red curve in Figure 2b) shifted to a lower energy compared to the commercial 2H-MoS_2_ (blue curve in Figure 2b), indicating that the valence state of Mo in *a*-PdP@1T′-MoS_2_ is lower than that of the commercial 2H-MoS_2_ due to the more electron-rich state on the 1T′-MoS_2_ surface.^19,44^ The local coordination environment of Mo in the MoS_2_ was investigated by the Fourier transform (FT) of EXAFS spectra in the R space (Figure 2c) and the fitting results (Table S1). Compared to the Mo–Mo bond distance of 3.17 Å in 2H-MoS_2_, a significantly shorter Mo–Mo bond distance of 2.77 Å is observed in the 1T′-MoS_2_ MLs on *a*-PdP NPs (Table S1), which could arise from the structural distortion characteristic of the 1T′ phase.^44^

**Figure 2:**
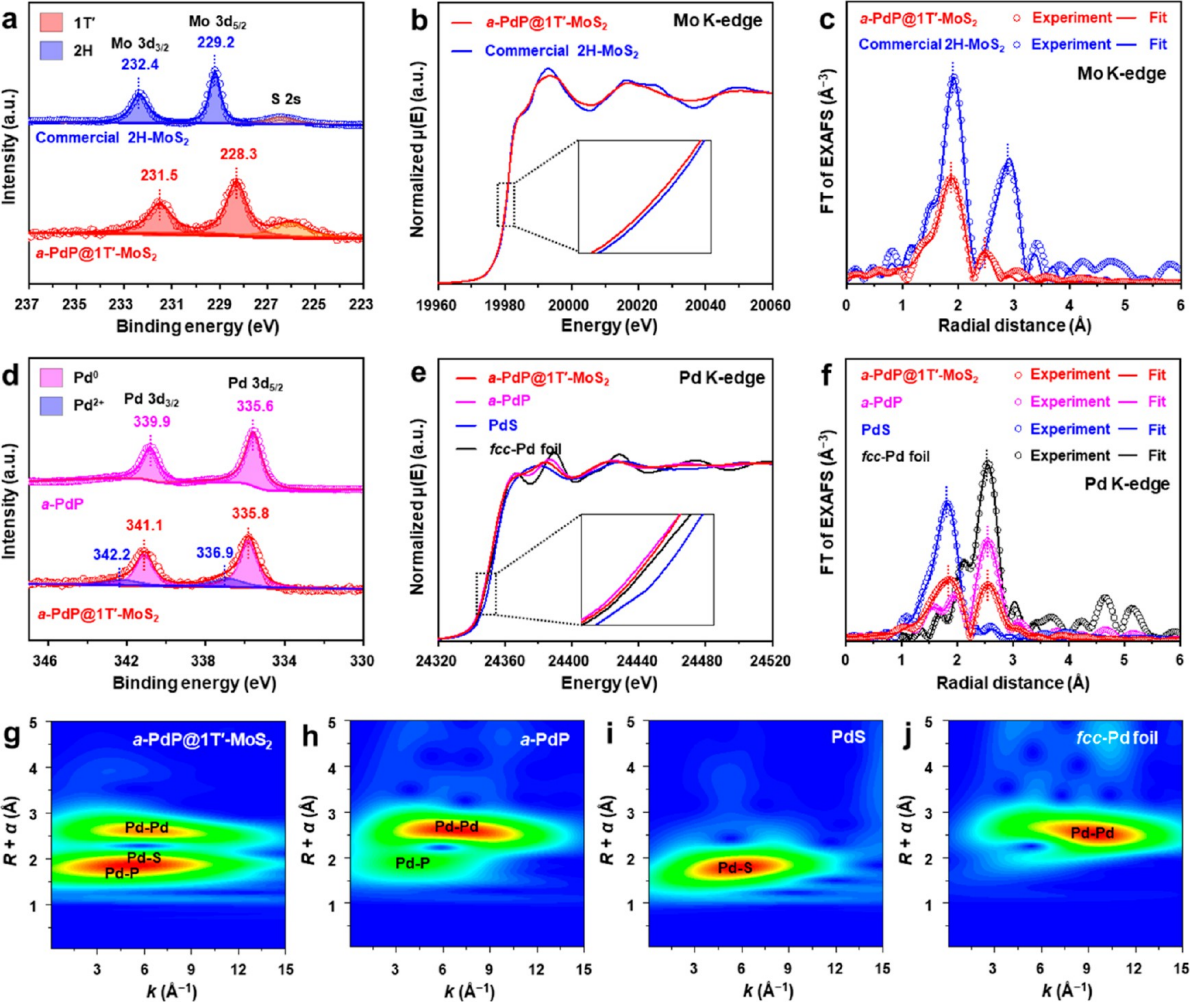
Characterization of electronic structure and coordination environment of the as-prepared *a*-PdP@1T′-MoS_2_ NPs. (a–c) High-resolution XPS Mo 3d spectra (a), Mo K-edge XANES spectra (b), and Fourier transformed Mo K-edge EXAFS spectra in the R space (c) of *a*-PdP@1T′-MoS_2_ NPs and commercial 2H-MoS_2_. (d) High-resolution XPS Pd 3d spectra of *a*-PdP@1T′-MoS_2_ NPs and *a*-PdP NPs. (e,f) Pd K-edge XANES spectra (e) and Fourier transformed Pd K-edge EXAFS spectra in the R space (f) of the as-prepared *a*-PdP@1T′-MoS_2_ NPs, *a*-PdP NPs, PdS, and *fcc*-Pd foil. (g–j) *k* ^2^-Weighted Pd K-edge wavelet transform (WT)-EXAFS contour plots of *a*-PdP@1T′-MoS_2_ NPs (g), *a*-PdP NPs (h), PdS (i), and *fcc*-Pd foil (j).

Moreover, we also investigated the electronic structure and coordination environment of Pd in the as-prepared *a*-PdP@1T′-MoS_2_ NPs, using the pristine *a*-PdP, crystalline PdS, and *fcc*-Pd foil as reference materials. XPS spectrum of *a*-PdP@1T′-MoS_2_ NPs (red curve in Figure 2d) shows two characteristic peaks located at 341.1 and 335.8 eV, which can be assigned to the Pd 3d_3/2_ and Pd 3d_5/2_ of metallic Pd, respectively.^38,41^ Both of them shift to a higher binding energy by ∼0.2 eV in comparison with Pd 3d_3/2_ (335.6 eV) and Pd 3d_5/2_ (335.8 eV) of *a*-PdP (pink curve in Figure 2d), indicating the electron transfer from *a*-PdP to 1T′-MoS_2_. Moreover, the presence of a small amount of Pd^2+^ (342.2 and 336.9 eV) in the *a*-PdP@1T′-MoS_2_ NPs indicates a slight sulfidation on the *a*-PdP surface during the growth of 1T′-MoS_2_ ML.^37,41^ The absorption edge position of *a*-PdP@1T′-MoS_2_ in the Pd K-edge XANES spectrum (red curve in Figure 2e) shifted to a lower energy compared to that of the PdS (blue curve in Figure 2e) and maintained a similar energy with the *fcc*-Pd foil (black curve in Figure 2e), indicating that the Pd in *a*-PdP@1T′-MoS_2_ NPs mainly maintains the metallic state after the growth of 1T′-MoS_2_ MLs. However, a slight shift to higher energy compared to the *a*-PdP (pink curve in Figure 2e) is observed, suggesting an electron transfer from the *a*-PdP to 1T′-MoS_2_, which is consistent with the XPS results (Figure 2d). The FT of EXAFS spectra in the R space (Figure 2f) and the fitting results (Table S2) are used to reveal the local structure of Pd in the *a*-PdP@1T′-MoS_2_. Compared to the bond lengths in the *a*-PdP (2.27 Å for Pd–P and 2.76 Å for Pd–Pd, pink curve in Figure 2f), a new bond with a bond length of 2.38 Å can be observed in the *a*-PdP@1T′-MoS_2_ (red curve in Figure 2f), which is in good accordance with the Pd–S bond distance in PdS (2.31 Å, blue curve in Figure 2f), indicating the existence of the strong interaction of Pd and S, i.e., the formation of Pd–S bonds, between *a*-PdP NPs and 1T′-MoS_2_ MLs. Furthermore, the presence of Pd–Pd, Pd–P, and Pd–S bonds in the synthesized *a*-PdP@1T′-MoS_2_ can be directly evidenced by its wavelet-transform (WT)-EXAFS contour plots (Figure 2g), as compared with the reference samples, i.e., *a*-PdP (Figure 2h), PdS (Figure 2i), and *fcc*-Pd foil (Figure 2j).

Importantly, the aforementioned wet-chemical method is general and robust, which has also been used to synthesize the high-phase-purity 1T′-WS_2_ and 1T′-MoWS_2_ MLs on the *a*-PdP NPs to form the *a*-PdP@1T′-WS_2_ core–shell NPs (Figure 3a–e, Note S1, Figure S8–S11, Table S3) and *a*-PdP@1T′-MoWS_2_ core–shell NPs (Figure 3f–j, Note S2, Figure S12–S15, Tables S1 and S3), respectively.

**Figure 3:**
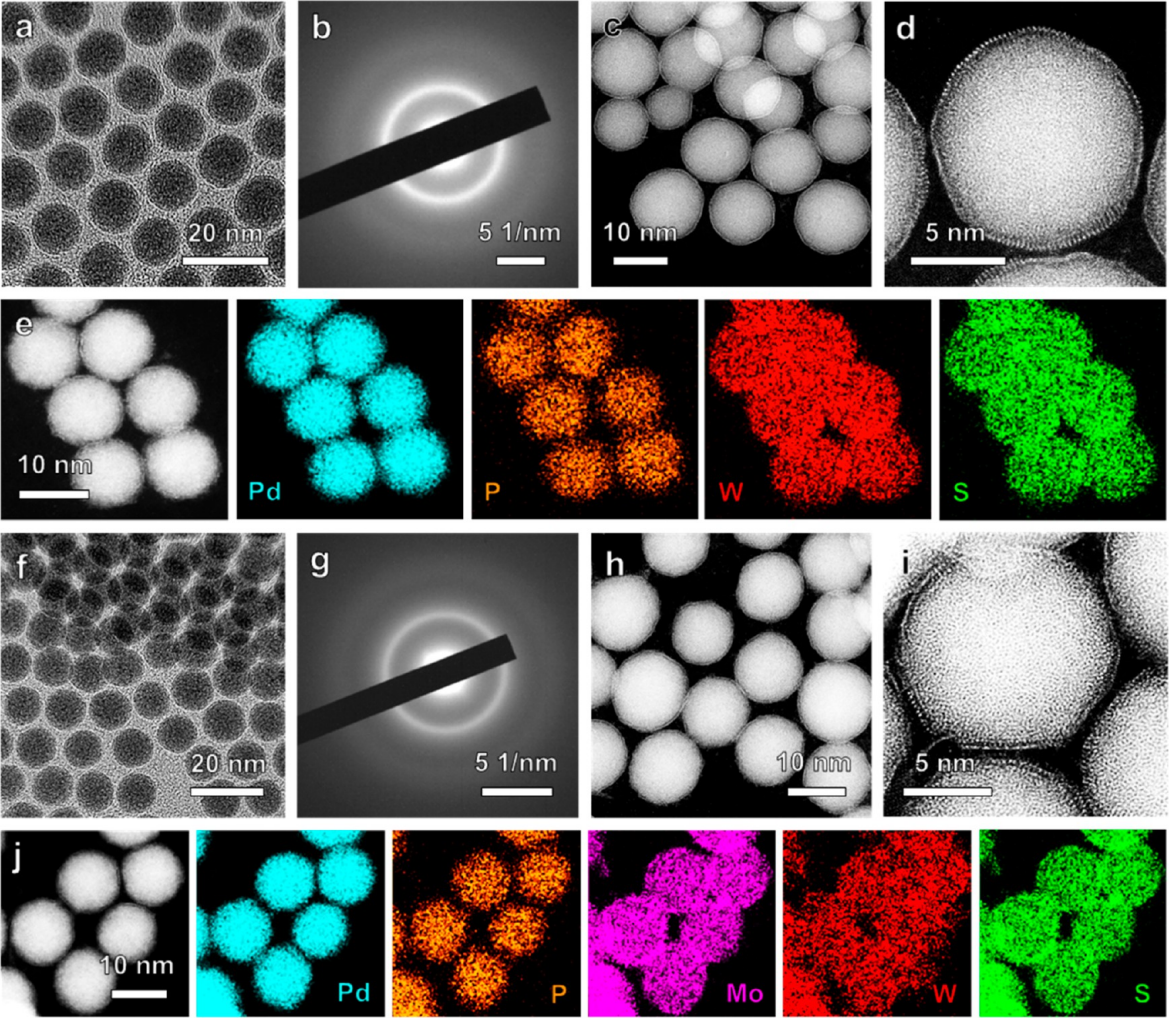
Characterization of the as-prepared *a*-PdP@1T′-WS_2_ and *a*-PdP@1T′-MoWS_2_ NPs. (a–c) Low-magnification TEM image (a), SAED pattern (b), and HAADF-STEM image (c) of *a*-PdP@1T′-WS_2_ NPs. (d) Atomic-resolution HAADF-STEM image of a representative *a*-PdP@1T′-WS_2_ NP. (e) STEM image and the corresponding EDS elemental maps of *a*-PdP@1T′-WS_2_ NPs. (f–h) Low-magnification TEM image (f), SAED pattern (g), and HAADF-STEM image (h) of *a*-PdP@1T′-MoWS_2_ NPs. (i) Atomic-resolution HAADF-STEM image of a representative *a*-PdP@1T′-MoWS_2_ NP. (j) STEM image and the corresponding EDS elemental maps of *a*-PdP@1T′-MoWS_2_ NPs.

### 2.2 Formation and Stabilization of 1T′-MoS_2_ MLs on *a*-PdP NPs

To explain the formation and stabilization of 1T′-MoS_2_ MLs on *a*-PdP NPs, we first conducted an ab initio molecular dynamics (AIMD) simulation to construct the *a*-PdP (Figure S16a), *a*-PdP@1T′-MoS_2_ (Figure S16b), and *a*-PdP@1H-MoS_2_ (1H-MoS_2_ means the ML of 2H-MoS_2_, Figure S16c) structures (see Supporting Information for details). Then, we performed density functional theory (DFT) calculations to evaluate the binding energies between the *a*-PdP and MoS_2_ MLs. The results show that the binding energy between the 1T′-MoS_2_ ML and *a*-PdP is 0.93 eV·f.u.^–1^, which is stronger than that between 1H-MoS_2_ and *a*-PdP (0.84 eV·f.u.^–1^), indicating a relatively stronger interaction between 1T′-MoS_2_ and *a*-PdP (Figure S17). The stronger interaction is further corroborated by the charge density difference analysis, which reveals a greater charge density disturbance for 1T′-MoS_2_ on *a*-PdP (Figure 4a) compared to that of 1H-MoS_2_ on *a*-PdP (Figure 4b). This is quantitatively supported by Bader charge analysis, which shows a significantly larger electron transfer from *a*-PdP to 1T′-MoS_2_ (2.11 e, Figure 4a) than that from *a*-PdP to 1H-MoS_2_ (1.28 e, Figure 4b). To further elucidate the binding difference at the Pd/S interface, we performed a crystal orbital Hamiltonian population (COHP) analysis, in which the bonding and antibonding interactions give negative and positive COHP values, respectively, and the integrated COHP (ICOHP) up to the Fermi level provides an estimation of bond strength. Figure 4c is the Pd–S bonding analysis concerning the bond length and ICOHP. In the relaxed model, the interface between 1T′-MoS_2_ and *a*-PdP has relatively more and shorter Pd–S bonds than that between 1H-MoS_2_ and *a*-PdP. Moreover, the ICOHP value for the 1T′-MoS_2_ is generally lower than that for 1H-MoS_2_ at the same bond length, indicating that the Pd–S bond strength in *a*-PdP@1T′-MoS_2_ is slightly stronger than that in the *a*-PdP@1H-MoS_2_. A complete COHP plot considering all Pd–S bonds is given in Figure 4d.

**Figure 4:**
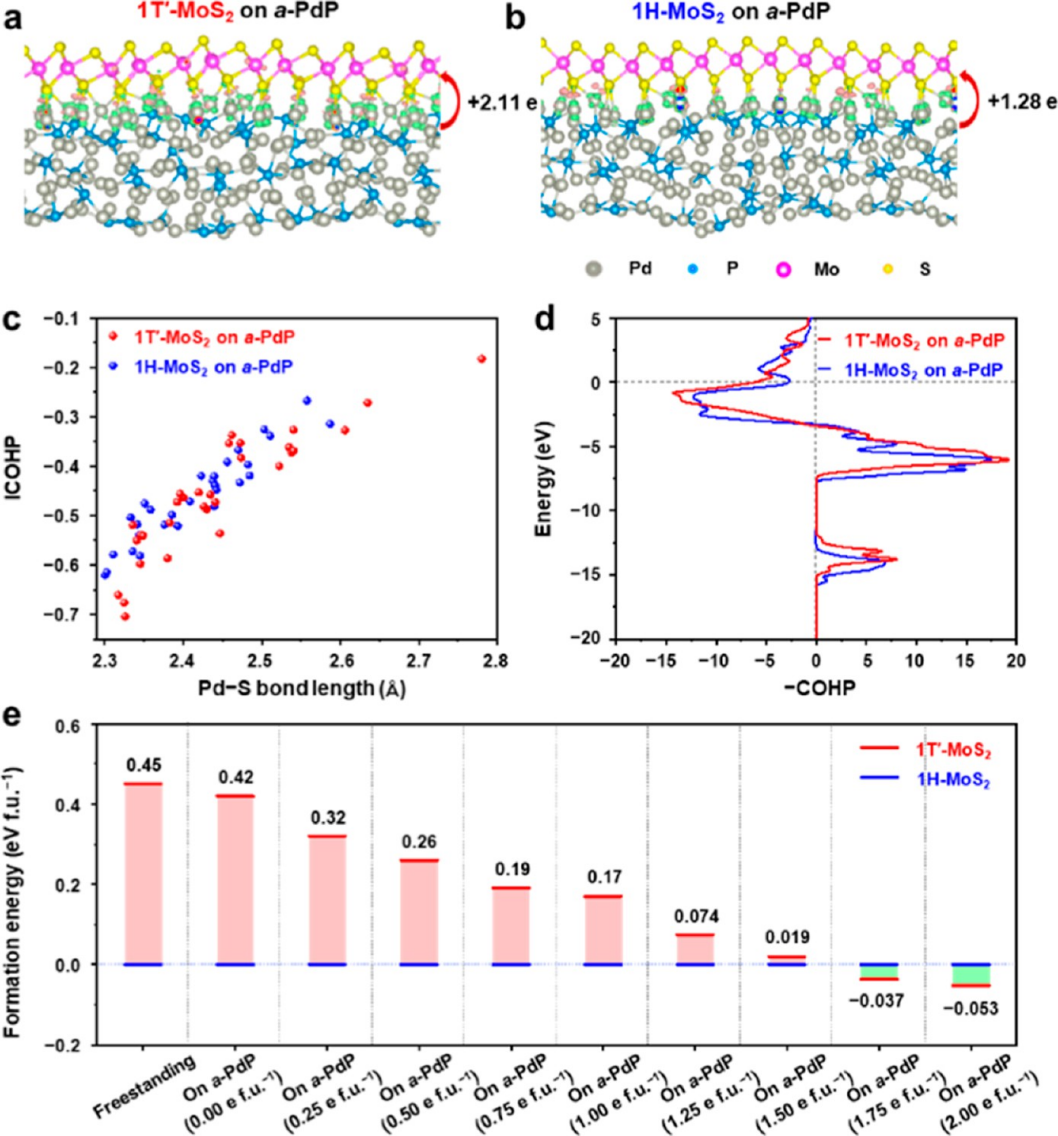
Formation and stabilization mechanism of 1T′-MoS_2_ MLs on *a*-PdP NPs. (a,b) Side views of charge-density differences of 1T′-MoS_2_ ML on *a*-PdP (a), and 1H-MoS_2_ on *a*-PdP (b). The green and red colors indicate the electron depletion and accumulation zones, respectively. The red curved arrows indicate the charge transfer from *a*-PdP to MoS_2_ MLs. The gray, blue, pink, and golden balls represent Pd, P, Mo, and S atoms, respectively. (c) Analyses of Pd–S bonds for 1H-MoS_2_ and 1T′-MoS_2_ on the *a*-PdP surface. (d) The COHP value of all Pd–S bonds for 1H-MoS_2_ and 1T′-MoS_2_ on *a*-PdP. The Fermi level is shifted to 0 eV as an energy reference. (e) The formation energy difference between 1T′-MoS_2_ and 1H-MoS_2_ on *a*-PdP. The formation energy of 1H-MoS_2_ is set as the energy reference. The label of “on *a*-PdP (n e f.u.^–1^)” signifies that each formula unit of MoS_2_ on the *a*-PdP substrate is charged with n electrons, where n ranges from 0.00 to 2.00 in steps of 0.25.

Our DFT calculations reveal that the freestanding 1H-MoS_2_ is more energetically favorable than the 1T′-MoS_2_, with a formation energy difference of 0.45 eV per formula unit (eV f.u.^–1^) between 1T′-MoS_2_ and 1H-MoS_2_ (Figure 4e). However, by using *a*-PdP as a template, this formation energy difference slightly decreases from 0.45 eV·f.u.^–1^ to 0.42 eV·f.u.^–1^ (Figure 4e). This result demonstrates the significant role of the strong Pd–S interaction in the formation and stabilization of 1T′-MoS_2_ MLs on *a*-PdP NPs, which is consistent with the experimental results (Figure 2). Moreover, the solution effect also plays an important role in stabilizing 1T′-MoS_2_ on the *a*-PdP. Under our experimental condition, MoS_2_ is surrounded by an oleylamine solution, which often acts as a charge donor and may further stabilize the electrophilic 1T′-MoS_2_.^44^ On the *a*-PdP surface, the calculated formation energy difference between 1T′-MoS_2_ and 1H-MoS_2_ decreases with the charge doping. In particular, when the charge doping exceeds 1.5 e f.u.^–1^, the 1T′-MoS_2_ on *a*-PdP becomes more stable than the 1H-MoS_2_.

To further confirm the role of oleylamine in synthesizing and stabilizing the 1T′-MoS_2_ MLs on *a*-PdP NPs, additional control experiments were conducted, i.e., oleylamine was mixed with oleic acid during the synthesis of *a*-PdP@MoS_2_ NPs. As shown in Figure S18, the Raman and XPS spectra indicate that the phase purity of the 1T′ phase gradually decreases from 100% to 75%, 59%, and 18% as the volume ratio of oleylamine and oleic acid is reduced successively from 3.0:0.0 to 2.5:0.5, 1.5:1.5, and 0.5:2.5.

Therefore, the high stability of 1T′-MoS_2_ synthesized on *a*-PdP via our wet-chemical method might arise from the strong Pd–S interaction and charge doping from the oleylamine in solution.

### 2.3 Electrocatalytic HER Performance of *a*-PdP@1T′-MoS_2_ NPs

The HER performance of the as-prepared *a*-PdP@1T′-MoS_2_ catalyst (Figure 1) was evaluated in a three-electrode system with N_2_-saturated 0.5 M H_2_SO_4_ as the electrolyte (see Supporting Information for details). For comparison, the pristine *a*-PdP NPs (Figure S1), and the commercial Pd/C and Pt/C catalysts, were also tested under the same conditions. The HER polarization curves of these catalysts were recorded at a scan rate of 5 mV·s^–1^ (Figure 5a) and performed on three independent batches of experiments (Figure S19). As shown in Figure 5a,b, the *a*-PdP@1T′-MoS_2_ NPs achieve current densities of 10, 500, and 1,000 mA·cm^–2^ at low overpotentials of only −35.8, −132.4, and −182.3 mV, respectively, which are lower than that of *a*-PdP NPs (−66.9, −420.2, and −669.9 mV, respectively), and commercial Pd/C (−63.3, −641.0, and −1,155.9 mV, respectively). Although the *a*-PdP@1T′-MoS_2_ catalyst displays a higher overpotential than the benchmark commercial Pt/C (−17.5 mV) at 10 mA·cm^–2^, it outperforms the Pt/C at current densities of 500 and 1,000 mA cm^–2^ (−168.1 and −310.9 mV, respectively), demonstrating its superior HER activity for high-current-density water electrolysis. As shown in Figure 5c, the Tafel slope of *a*-PdP@1T′-MoS_2_ is 27.1 mV·dec^–1^, which is lower than that of Pt/C (28.4 mV·dec^–1^), Pd/C (37.9 mV·dec^–1^), and *a*-PdP (59.6 mV·dec^–1^). This low Tafel slope value (27.1 mV·dec^–1^) indicates that the whole HER reaction in the *a*-PdP@1T′-MoS_2_ follows the Volmer–Tafel mechanism,^45,46^ demonstrating its rapid hydrogen recombination kinetics.

**Figure 5:**
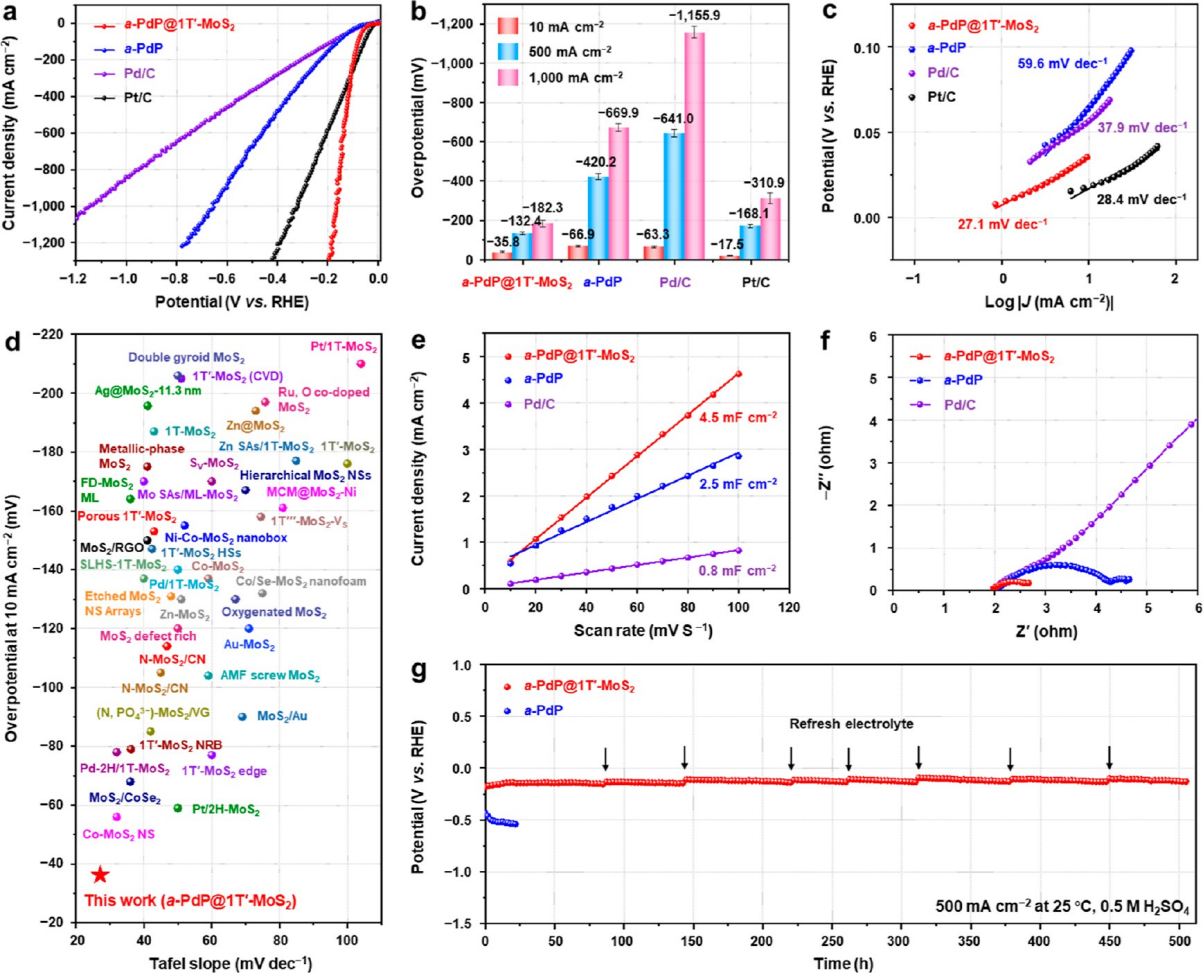
Electrocatalytic HER performance of *a*-PdP@1T′-MoS_2_ NPs. (a) HER polarization curves of *a*-PdP@1T′-MoS_2_ NPs, *a*-PdP NPs, commercial Pd/C, and commercial Pt/C recorded in N_2_-saturated 0.5 M H_2_SO_4_ electrolyte at a scan rate of 5 mV·s^–1^. (b) Comparison of the overpotentials of different HER catalysts at the current densities of 10, 500, and 1,000 mA·cm^–2^. The error bar represents the standard deviation (SD). (c) Tafel plots for HER obtained from the corresponding polarization curves in (a). (d) Comparison of the overpotentials at 10 mA·cm^–2^, and Tafel slopes of *a*-PdP@1T′-MoS_2_ NPs and most previously reported representative MoS_2_-based electrocatalysts for HER in 0.5 M H_2_SO_4_. (e) Plot of capacitive current density versus scan rate. The electrochemical double-layer capacitances (*C*_dl_), measured on *a*-PdP@1T′-MoS_2_ NPs, *a*-PdP NPs, and commercial Pd/C, correspond to the slopes of the linear fits to their data. (f) The EIS Nyquist plots of *a*-PdP@1T′-MoS_2_ NPs, *a*-PdP NPs, and commercial Pd/C. (g) Chronopotentiometry tests at 500 mA·cm^–2^ of the *a*-PdP@1T′-MoS_2_ NPs and *a*-PdP NPs. The electrolyte was refreshed at time marked by black arrows.

In addition, we evaluated the HER performance of the as-prepared *a*-PdP@1T′-TMD catalysts. As shown in Figure S20, *a*-PdP@1T′-MoS_2_ outperforms *a*-PdP@1T′-MoWS_2_ and *a*-PdP@1T′-WS_2_. All of the aforementioned results place our *a*-PdP@1T′-MoS_2_ catalyst among the best reported MoS_2_-based catalysts toward HER (Figure 5d, Table S4).

The electrochemically active surface areas (ECSAs) of the catalysts for the HER were calculated based on the value of the electrochemical double-layer capacitance (*C*_dl_) obtained from cyclic voltammetry (CV) measurements at various scan rates (Figure S21). The *C*_dl_ (Figure 5e) and ECSA (Figure S22a) values of *a*-PdP@1T′-MoS_2_ are calculated to be 4.5 mF·cm^–2^ and 28.13 cm^2^, respectively, higher than those of *a*-PdP (2.5 mF·cm^–2^ and 15.63 cm^2^, respectively) and Pd/C (0.8 mF·cm^–2^ and 5.00 cm^2^, respectively), indicating more exposed active sites in the *a*-PdP@1T′-MoS_2_ catalyst. To account for the different amounts of active sites, we further normalized the HER activities by ECSAs. As shown in Figure S22b, the *a*-PdP@1T′-MoS_2_ exhibits the highest *C*_dl_-normalized HER activity compared to the *a*-PdP and commercial Pd/C, further confirming its superior intrinsic catalytic performance. Furthermore, electrochemical impedance spectroscopy (EIS) reveals that the *a*-PdP@1T′-MoS_2_ displays a charge transfer resistance (*R*_ct_) of 0.7 Ω, lower than that of *a*-PdP (2.3 Ω) and commercial Pd/C (Figure 5f), suggesting more efficient charge transfer on the *a*-PdP@1T′-MoS_2_ during HER.

The long-term electrochemical stabilities of *a*-PdP@1T′-MoS_2_ and the reference catalyst (*a*-PdP) were evaluated via a chronopotentiometry test at a current density of 500 mA·cm^–2^ (Figure 5g). Impressively, *a*-PdP@1T′-MoS_2_ exhibits exceptional stability, maintaining its initial overpotential with negligible decay for over 500 h. In contrast, *a*-PdP, which requires higher overpotentials, undergoes a rapid degradation within a few hours. The characterizations of *a*-PdP@1T′-MoS_2_ NPs, including the morphology, crystal phase, and electronic structure, after the HER durability test confirm that the exceptional stability of the *a*-PdP@1T′-MoS_2_ catalyst originates from its unique architecture, i.e., the *a*-PdP NPs are uniformly encapsulated by the 1T′-MoS_2_ MLs, effectively stabilizing both the amorphous phase of PdP NPs and the 1T′ phase of MoS_2_ MLs (Figure S23). However, it is worth mentioning that while maintaining the 1T′ phase, the MoS_2_ MLs exhibit a few defects during the long-term durability test, leading to partial exposure of the *a*-PdP surface (Figure S23d). In contrast, the poor HER stability of the pristine *a*-PdP NPs is attributed to the damage and inevitable amorphous-to-crystalline phase transition of the *a*-PdP NPs after the HER durability test (Figure S24).

The superior HER performance of the *a*-PdP@1T′-MoS_2_ catalyst can be attributed to the following reasons. First, MoS_2_ exhibits phase-dependent HER activity, i.e., the semimetallic 1T′-MoS_2_ demonstrates superior catalytic performance to the semiconducting 2H-MoS_2_, owing to its enhanced basal-plane activity and higher electrical conductivity.^19,20^ Second, a few defects in the 1T′-MoS_2_ MLs during the long-term durability test could lead to partial exposure of the *a*-PdP surface, which could further enhance the HER performance. As reported previously, amorphous nanostructures possess abundant unsaturated coordination sites and dangling bonds, arising from their disordered atomic arrangement, which endow them with catalytic performance surpassing that of their crystalline counterparts.^35,37^ Third, in the metal@2D material core–shell structures, the electrons from the metal core could penetrate the 2D material shell, thereby optimizing the electronic structure of the 2D material shell (e.g., Fermi level position, density of states at the Fermi level, and surface work function), facilitating the adsorption and reaction of molecules preferentially on the 2D material surface.^47^ Projected density of states (PDOS) calculations (Figure S25) reveal a significant downshift in the *p*-band center (ε_*p*_) of S atoms from −1.12 eV in the pristine 1T′-MoS_2_ to −1.65 eV in the *a*-PdP@1T′-MoS_2_, resulting in a weaker hydrogen adsorption on the S atoms of the *a*-PdP@1T′-MoS_2_. Furthermore, the reduced energy gap near the Fermi level in the *a*-PdP@1T′-MoS_2_ contributes to its enhanced electrical conductivity compared to the pristine 1T′-MoS_2_. Additionally, in our *a*-PdP@1T′-MoS_2_, the electron transfer from *a*-PdP to 1T′-MoS_2_, which is also supported by the charge-density difference of 1T′-MoS_2_ ML on the *a*-PdP surface (Figure 4a), and the resultant electronic structure optimization are revealed by XPS (Figure 2d) and XANES (Figure 2e) analyses. As known, 2D materials normally exhibit thickness-dependent catalytic behavior. The atomically thin 2D nanosheets show superior HER performance compared to their bulk counterparts.^9,10^ In the metal@2D material core–shell structures, the thickness of 2D materials could affect the electron penetration efficiency from the metal core through the 2D material shell. A thinner 1T′-MoS_2_ shell enables more efficient electron transfer and thus higher HER activity.^48,49^ Therefore, the 1T′-MoS_2_ MLs in *a*-PdP@1T′-MoS_2_ could achieve an optimal electron transfer efficiency and catalytic activity. Fourth, the biaxial strain introduced by the highly curved surface of *a*-PdP NPs can significantly enhance HER performance, owing to its ability to generate surface defects, intensify the electronic field, and optimize the adsorption configurations of reactants on 1T′-MoS_2_ MLs.^50,51^ Fifth, the 1T′-MoS_2_ shell remains highly stable under harsh conditions, such as strong acidic environments, which can also effectively encapsulate and protect the *a*-PdP core from degradation. Therefore, the design of the *a*-PdP@1T′-MoS_2_ core–shell structure can simultaneously address the key challenges of activity and stability during the acidic HER, offering a distinct advantage.

### 2.4 Synthesis and Characterization of *s*-Pt/*a*-PdP@1T′-MoS_2_ NPs

To further enhance the HER performance, our *a*-PdP@1T′-MoS_2_ was used as a template to grow *s*-Pt via electrochemical deposition (Figure 6a, see Supporting Information for details). The successful formation of *s*-Pt was confirmed by atomic-resolution HAADF-STEM, in which the Pt atoms, marked by the orange dashed circles in Figure 6b, exhibit a significantly brighter contrast than the Mo atoms. The STEM-EDS elemental maps verify the homogeneous distribution of Pt on the *a*-PdP@1T′-MoS_2_ (Figure 6c), and the Pt loading up to 2.3 wt% is determined by the STEM-EDS spectrum (Figure S26).

**Figure 6:**
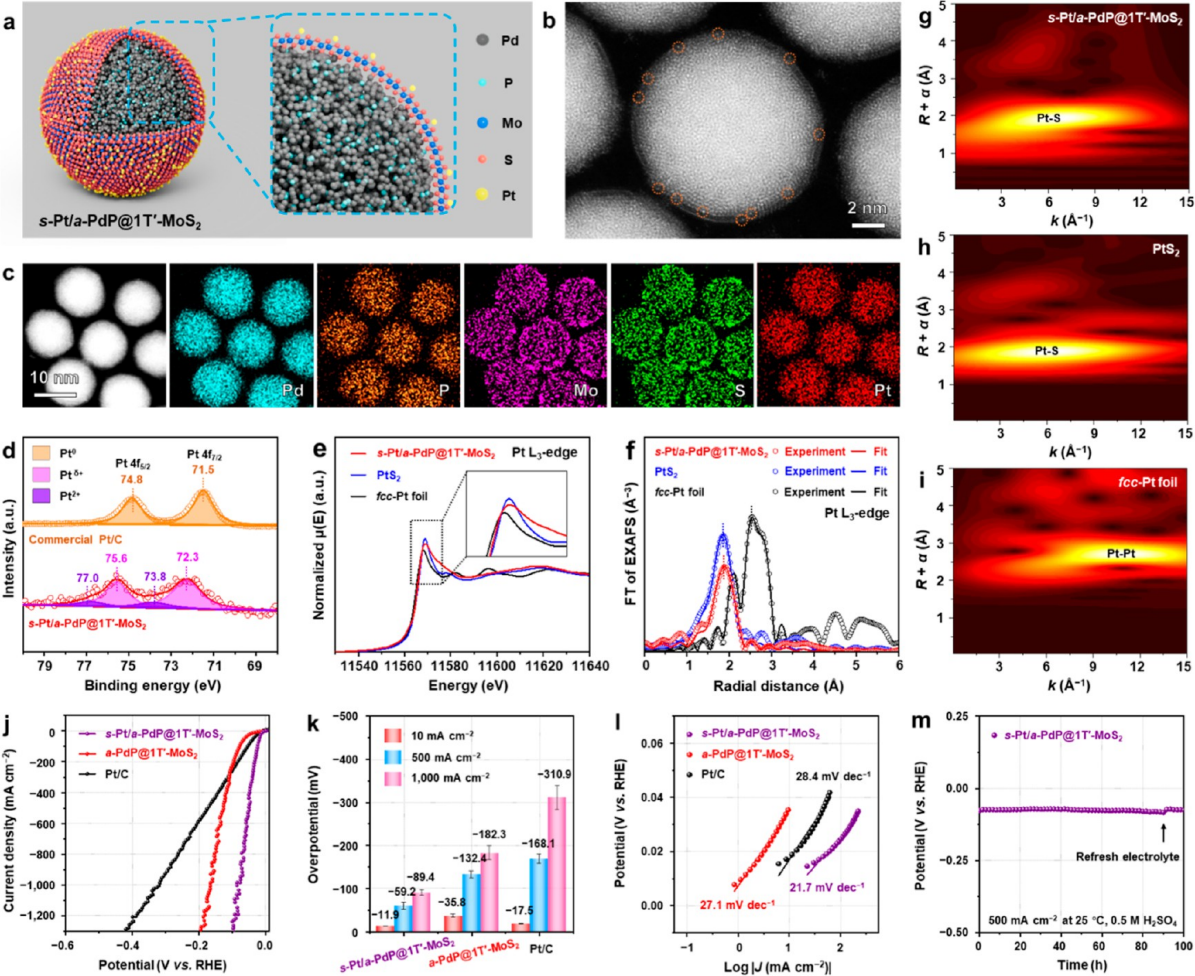
Synthesis and characterization of *s*-Pt/*a*-PdP@1T′-MoS_2_ NPs. (a,b) Schematic illustration (a) and atomic-resolution HAADF-STEM image (b) of a representative *s*-Pt/*a*-PdP@1T′-MoS_2_ NP, showing the isolated *s*-Pt on the *a*-PdP@1T′-MoS_2_. (c) STEM image and the corresponding EDS elemental maps of *s*-Pt/*a*-PdP@1T′-MoS_2_ NPs. (d) High-resolution XPS Pt 4f spectra of *s*-Pt/*a*-PdP@1T′-MoS_2_ NPs and commercial Pt/C. (e,f) Pt L_3_-edge XANES spectra (e) and Fourier transformed Pt L_3_-edge EXAFS spectra in the R space (f) of *s*-Pt/*a*-PdP@1T′-MoS_2_ NPs, PtS_2_, and *fcc*-Pt foil. (g–i) *k* ^2^-Weighted Pt L_3_-edge wavelet transform (WT)-EXAFS contour plots of *s*-Pt/*a*-PdP@1T′-MoS_2_ NPs (g), PtS_2_ (h), and *fcc*-Pt foil (i). (j) HER polarization curves of *s*-Pt/*a*-PdP@1T′-MoS_2_, *a*-PdP@1T′-MoS_2_, and commercial Pt/C recorded in N_2_-saturated 0.5 M H_2_SO_4_ electrolyte at a scan rate of 5 mV·s^–1^. (k) Comparison of the overpotentials of different HER catalysts, i.e., *s*-Pt/*a*-PdP@1T′-MoS_2_, *a*-PdP@1T′-MoS_2_, and commercial Pt/C, at the current densities of 10, 500, and 1,000 mA·cm^–2^, respectively. The error bar represents the SD (l) Tafel plots for HER obtained from the corresponding polarization curves in (j). (m) Chronopotentiometry tests at 500 mA·cm^–2^ of the *s*-Pt/*a*-PdP@1T′-MoS_2_. The electrolyte was refreshed at time marked by the black arrow.

The chemical state of Pt in the *s*-Pt/*a*-PdP@1T′-MoS_2_ NPs was investigated by XPS (Figure 6d). Compared to the commercial Pt/C with two peaks located at 71.5 (Pt 4f_7/2_) and 74.8 eV (Pt 4f_5/2_), which can be assigned to the metallic Pt, the Pt 4f spectrum of *s*-Pt/*a*-PdP@1T′-MoS_2_ was fitted with two doublets at higher binding energies, i.e., 72.3­(Pt^δ+^)/73.8­(Pt^2+^) and 75.6­(Pt^δ+^)/77.0­(Pt^2+^) eV, respectively.^21,52^ The observed positive shift suggests the electron transfer from Pt to the neighboring S, confirming the formation of Pt–S bonds. XANES and EXAFS were used to confirm the electronic and coordination structure of the *s*-Pt in *s*-Pt/*a*-PdP@1T′-MoS_2_. As shown in Figure 6e, the white line intensity at the Pt L_3_-edge for *s*-Pt/*a*-PdP@1T′-MoS_2_ is closer to the PtS_2_, but much higher than that of the *fcc*-Pt foil, indicating a dominantly higher chemical valence state of the Pt species. The corresponding FT of EXAFS spectra in the R space (Figure 6f) show a primary peak at 1.89 Å, assigned to the Pt–S bonds, and the quantitative fitting indicates an average coordination number of 4.0 and an average bond length of 2.32 Å (Table S5). The absence of a metallic Pt–Pt scattering path confirms the single-atomic dispersion of Pt on the *s*-Pt/*a*-PdP@1T′-MoS_2_.^53,54^ As shown in Figure 6f, there is no peak attributable to the metallic Pt–Pt bond, confirming the *s*-Pt on the *a*-PdP@1T′-MoS_2_. This result is further supported by the WT-EXAFS analysis (Figure 6g–i), in which the contour plot of *s*-Pt/*a*-PdP@1T′-MoS_2_ (Figure 6g) is quite similar to that of PtS_2_ (Figure 6h) but significantly different from that of the *fcc*-Pt foil (Figure 6i).

Moreover, the HER performance of the as-prepared *s*-Pt/*a*-PdP@1T′-MoS_2_ NPs was evaluated. As shown in Figure 6j,k, the *s*-Pt/*a*-PdP@1T′-MoS_2_ NPs achieve current densities of 10, 500, and 1,000 mA·cm^–2^ at low overpotentials of only −11.9, −59.2, and −89.4 mV, respectively, which are lower than that of *a*-PdP@1T′-MoS_2_ NPs (−35.8, −132.4, and −182.3 mV, respectively) and commercial Pt/C (−17.5, −168.1, and −310.9 mV, respectively), demonstrating superior HER activity and promising potential for high-current-density water electrolysis. As shown in Figure 6l, the Tafel slope of *s*-Pt/*a*-PdP@1T′-MoS_2_ is 21.7 mV·dec^–1^, which is lower than that of *a*-PdP@1T′-MoS_2_ (27.1 mV·dec^–1^) and Pt/C (28.4 mV·dec^–1^), suggesting its faster HER reaction kinetics. As shown in Figure 6m, *s*-Pt/*a*-PdP@1T′-MoS_2_ exhibits exceptional stability, maintaining its initial overpotential with negligible decay for over 100 h. Impressively, our *s*-Pt/*a*-PdP@1T′-MoS_2_ catalyst demonstrates superior HER performance compared to the previously reported single-atomically dispersed Pt catalysts (Table S6).

To elucidate the electron transfer between *s*-Pt and *a*-PdP@1T′-MoS_2_, we performed DFT calculations to analyze the charge density difference (Figure S27). The result reveals significant charge accumulation around the *s*-Pt atoms. This observation is quantitatively corroborated by Bader charge analysis, which indicates an electron transfer of 0.35 e from the S atom in *a*-PdP@1T′-MoS_2_ to the *s*-Pt. This electron enrichment of *s*-Pt on the support, consistent with previous reports, is known to facilitate faster HER kinetics.^55–57^

To further elucidate the HER activity of *s*-Pt/*a*-PdP@1T′-MoS_2_, DFT calculations were conducted based on the established models of distinct active sites (Figure S28). The Gibbs free energy for hydrogen adsorption (Δ*G*_H_) serves as a widely accepted descriptor for intrinsic HER activity, in which the catalytic site achieving a |Δ*G*_H_| value close to zero is considered optimal according to the Sabatier principle.^58,59^ The calculated Δ*G*_H_ diagrams of S sites on *a*-PdP@1T′-MoS_2_ and 1T′-MoS_2_, Pd sites on *a*-PdP@1T′-MoS_2_ and *a*-PdP, and Pt sites on *s*-Pt/*a*-PdP@1T′-MoS_2_ and Pt(111), denoted as *a*-PdP@1T′-MoS_2_-(S), 1T′-MoS_2_-(S), *a*-PdP@1T′-MoS_2_-(Pd), *a*-PdP-(Pd), *s*-Pt/*a*-PdP@1T′-MoS_2_-(Pt), and Pt(111)-(Pt), respectively, are shown in Figure S29. The |Δ*G*_H_| values of *a*-PdP@1T′-MoS_2_-(S) (0.04 eV) and *a*-PdP@1T′-MoS_2_-(Pd) (0.15 eV) are lower than those of 1T′-MoS_2_-(S) (0.21 eV) and *a*-PdP-(Pd) (0.48 eV), respectively. These results demonstrate the crucial role of the interfacial interaction between *a*-PdP and 1T′-MoS_2_ in optimizing the proton binding strength on *a*-PdP@1T′-MoS_2_, thereby enhancing HER activity. Moreover, the |Δ*G*_H_| value of *s*-Pt/*a*-PdP@1T′-MoS_2_-(Pt) (−0.02 eV) is closer to 0 eV, lower than that of *a*-PdP@1T′-MoS_2_-(S) (0.04 eV) and Pt(111) (−0.31 eV), which could enable faster hydrogen adsorption and product release on the *s*-Pt site of *s*-Pt/*a*-PdP@1T′-MoS_2_.

These findings indicate that the *a*-PdP@1T′-MoS_2_ NPs can serve as an effective support for anchoring single-atom-dispersed metals, demonstrating great potential for catalytic applications.

## 3 Conclusions

In conclusion, we have developed a facile wet-chemical strategy to synthesize *a*-PdP@1T′-MoS_2_ core–shell NPs using *a*-PdP NPs as templates. The formation and stabilization of the high phase-purity 1T′ phase can be attributed to the strong Pd–S interaction, electron donation from oleylamine, and the unique amorphous nature of the *a*-PdP template, as confirmed by systematic characterization and theoretical calculations. The resulting *a*-PdP@1T′-MoS_2_ catalyst exhibits superior HER performance, achieving 1,000 mA·cm^–2^ at a low overpotential of −182.3 mV and maintaining remarkable stability for over 500 h. This study establishes a viable pathway for synthesizing high phase-purity, stable 1T′-TMD MLs on amorphous templates. It also demonstrates the promise of *a*-PdP@1T′-TMD core–shell heterostructures as highly efficient and stable platforms for anchoring single-atomically dispersed metals for other catalytic reactions beyond HER (e.g., *s*-Pt for oxygen reduction reaction (ORR), *s*-Cu for the CO_2_ reduction reaction (CO_2_RR), *s*-Fe for the nitrate reduction reaction (NH_3_RR), etc.), opening opportunities for the design and preparation of highly efficient electrocatalysts.

## Supplementary Material



## Notes

Chemicals, detailed experimental methods, characterizations, electrochemical measurements, computational details, additional figures, and tables (PDF)
