# Supplementary material for: Unconventional-Phase 1T′-Transition Metal Dichalcogenide Monolayers Grown on Amorphous Templates for Highly Efficient Hydrogen Evolution
Source: J Am Chem Soc. 2026 Feb 10;148(7):7342–54. doi: 10.1021/jacs.5c19857 (PMC12951452; doi:10.1021/jacs.5c19857)
Supplement: Supplementary file 1 [file ja5c19857_si_001.pdf]

Supporting Information

Unconventional-Phase 1T′-Transition Metal Dichalcogenide Monolayers Grown on Amorphous Templates for Highly Efficient Hydrogen Evolution

Zijian Li[1†], Hua Yang[1,2†], Mingjun Sun[3†], An Zhang[1†], Yiyao Ge[4], Xinyue Long[1], Biao Huang[1], Li Zhai[1], Wei Zhai[1], Lujiang Li[1], Lixin Wang[1], Chao Wang[1], Yanping Xu[1], Yanming Cai[1], Peigen Liu[5], Bo Chen[6], Lin Gu[7], Panzhe Qiao[8]*, Qinghua Zhang[9]*, Feng Ding[10]*, Hua Zhang[1,11,12,13]*

[1]Department of Chemistry, City University of Hong Kong, Hong Kong, China

[2]The Analysis & Testing Center, Beihang University, Beijing 102206, China

[3]Institute of Technology for Carbon Neutrality, Shenzhen Institute of Advanced Technology, Chinese Academy of Sciences, Shenzhen 518055, China

[4]State Key Laboratory for Advanced Metals and Materials, University of Science and Technology Beijing, Beijing 100083, China

[5]National Synchrotron Radiation Laboratory, University of Science and Technology of China, Hefei 230029, China

[6]State Key Laboratory of Flexible Electronics & Jiangsu Key Laboratory of Smart Biomaterials and Theranostic Technology, Institute of Advanced Materials, Nanjing University of Posts and Telecommunications, Nanjing 210023, China

[7]Beijing National Center for Electron Microscopy and Laboratory of Advanced Materials, Department of Materials Science and Engineering, Tsinghua University, Beijing 100084, China

[8]Shanghai Synchrotron Radiation Facility, Shanghai Advanced Research Institute, Chinese Academy of Sciences, Shanghai, 201210, China

[9]Beijing National Laboratory for Condensed Matter Physics, Institute of Physics, Chinese Academy of Sciences, Beijing 100190, China

[10]Research Division of Advanced Materials, Suzhou Laboratory, Suzhou 215133, China

[11]Hong Kong Institute for Clean Energy (HKICE), City University of Hong Kong, Kowloon, Hong Kong, China

[12]Hong Kong Branch of National Precious Metals Material Engineering Research Center (NPMM), City University of Hong Kong, Hong Kong, China

[13]Shenzhen Research Institute, City University of Hong Kong, Shenzhen, 518057, China

[†]These authors contributed equally to this work.

*Corresponding authors. E-mail: hua.zhang@cityu.edu.hk; dingf@szlab.ac.cn; zqh@iphy.ac.cn; qiaopz@sari.ac.cn

Methods section

Chemicals. Palladium(II) acetylacetonate ($Pd(acac)_2$ ≥99.9% trace metals basis), molybdenum(V) chloride ($MoCl_5$, 99.99% trace metals basis), tungsten (VI) chloride (WCl_6, ≥99.9% trace metals basis), sulfur powder (S, 99.98% trace metals basis), chloroplatinic(IV) acid hydrate ($H_2PtCl_6 \cdot xH_2O$, ≥99.9% trace metals basis), oleylamine (OAm, technical grade, 70%), 1-octadecene (ODE, technical grade, 90%), oleic acid (OA, technical grade, 90%), trioctylphosphine (TOP, technical grade, 90%), potassium hydroxide (KOH), sulfuric acid (H_2SO_4, technical grade), hydrochloric acid (technical grade), toluene (technical grade, ≥99%), and ethanol (technical grade) were purchased from Sigma-Aldrich. All chemicals were used as received without further purification. The Milli-Q water (resistivity of 18.2 MΩ·cm; Milli-Q System, Millipore) was used in our experiment.

Synthesis of amorphous P-doped Pd nanoparticles (*a*-PdP NPs). The *a*-PdP NPs were synthesized using a previously reported method[1] with slight modifications. Briefly, 40 mg of $Pd(acac)_2$, 20 mL of OAm (70%), 40 mL of ODE, and 2 mL of OA were degassed and stirred by heating at 100 °C under vacuum in a 100-mL three-necked flask. After degassing for 40 min, followed by the injection of 520 μL of TOP (90%). The temperature was then rapidly raised to 300 °C and maintained for 40 min. After naturally cooling to room temperature, the product was collected by centrifugation at 8,000 rpm for 5 min. The obtained *a*-PdP NPs with ~6 mg were purified by repeated washing with toluene and ethanol (three times each), and finally redispersed in toluene prior to further usage.

Synthesis of *a*-PdP@1T′-MoS$_2$ NPs. The *a*-PdP@1T′-MoS_2 NPs were synthesized using our method reported previously[2] with slight modifications. Briefly, 2 mL of OAm (70%) and 3 mL of ODE were added into a 50-mL three-necked flask and degassed under heating at 100 °C with continuous magnetic stirring. After 30 min of degassing, the atmosphere was replaced with Ar, and the temperature was rapidly raised to 280 °C. Concurrently, the following precursor solutions were prepared.

Solution A: 3 mL of the *a*-PdP toluene solution (containing 2 mg of *a*-PdP as determined by ICP-OES) were mixed with 2 mL of ethanol as a precipitant. After centrifugation at 8,000 rpm for 5 min, the precipitate was collected and then redispersed in 800 μL of OAm (70%).

Solution B: 9.6 mg of sulfur powder were dissolved in 1 mL of OAm (70%) by sonicating for 10 min.

Solution C: 6.84 mg of $MoCl_5$ were dissolved in 1 mL of OAm (70%) by sonicating for 10 min.

Subsequently, 180 μL of *Solution B* were mixed with *Solution A* to form *Solution D*.

Once the reaction temperature was stabilized at 280 °C, 180 μL of *Solution C* were injected into the flask, followed by the dropwise addition of *Solution D* over 2 min. After reaction for 10 min, heating was stopped prior to naturally cooling down to room temperature. The resulting *a*-PdP@1T′-MoS_2 NPs were then collected by centrifugation (8,000 rpm, 5 min), and dispersed in a mixture of ethanol and hydrochloric acid (v/v = 9/1). The obtained solution was continuously stirred under an Ar atmosphere for 10 min. After that, the *a*-PdP@1T′-MoS_2 NPs were collected by centrifugation (8,000 rpm, 5 min), and redispersed in ethanol for storage prior to usage for applications.

To demonstrate the scalability of our synthesis, we performed a large-scale reaction using 800 mg of $Pd(acac)_2$ and 10.4 mL of TOP (90%) in a mixed solution composed of 30 mL of OAm (70%), 60 mL of ODE and 3 mL of OA. The reaction was carried out at 300 °C for 40 min, yielding ~120 mg of *a*-PdP NPs in a single batch of experiment. Then, 1T′-MoS_2 MLs were grown on the as-synthesized *a*-PdP NPs (~120 mg) by reacting with 36.94 mg of $MoCl_5$ and 51.84 mg of sulfur powder in a mixed solution composed of 20 mL of OAm (70%) and 30 mL of ODE. The reaction was carried out at 280 °C for 10 min, yielding ~160 mg of *a*-PdP@1T′-MoS_2 NPs in a single batch of experiment (Figure S3).

Synthesis of *a*-PdP@1T′-WS_2 NPs. The procedures for the synthesis of *a*-PdP@1T′-

WS_2 NPs are same to that for the *a*-PdP@1T′-MoS_2 NPs, except that the *Solution C* was prepared by dissolving 9.92 mg of WCl_6 in 1 mL of OAm (70%) under 10-min sonication.

Synthesis of *a*-PdP@1T′-$MoWS_2$ NPs. The procedures for the synthesis of *a*-PdP@1T′-$MoWS_2$ NPs are same to that for the *a*-PdP@1T′-MoS_2 NPs, except that the *Solution C* was prepared by dissolving 3.42 mg of $MoCl_5$ and 4.96 mg of WCl_6 in 1 mL of OAm (70%) under 10-min sonication.

Synthesis of *s*-Pt/*a*-PdP@1T′-MoS_2 NPs. The *s*-Pt/*a*-PdP@1T′-MoS_2 NPs were prepared using a previously reported method[3] with slight modifications. Briefly, the single-atomically dispersed Pt (*s*-Pt) was deposited onto the *a*-PdP@1T′-MoS_2 *via* electrochemical deposition in a standard three-electrode cell. The carbon paper coated with the *a*-PdP@1T′-MoS_2 catalyst was directly used as working electrode, with an Ag/AgCl electrode as the reference electrode, and a carbon rod as the counter electrode (see **Preparation of electrodes** for details). The corresponding electrochemical deposition was carried out by multi-cycle cathode polarization in 1 M KOH solution containing 50 μM H_2PtCl_6 with a scan rate of 50 mV s^{-1} between 0 and −0.50 V versus reversible hydrogen electrode (RHE) for 200 cycles. After deposition, the as-prepared *s*-Pt/*a*-PdP@1T′-MoS_2 electrode was rinsed three times with deionized water, and used directly for subsequent electrochemical measurements.

Characterizations. Transmission electron microscopy (TEM) and dark-field scanning TEM (DF-STEM) images were recorded by using JEOL 2100F (Japan) operated at 200 kV. The high-angle annular DF-STEM (HAADF-STEM) images, annular bright-field STEM (ABF-STEM) images, and energy dispersive X-ray spectroscopy (EDS) results were obtained by JEOL ARM200F (JEOL, Tokyo, Japan) operated at 200 kV and equipped with double spherical aberration (Cs) correctors. Scanning electron microscope (SEM) images were recorded on a scanning electron microscope (Thermo Fisher Scientific, QUATTRO S). Rigaku SmartLab and Bruker D8 ADVANCE X-ray powder diffractometers with Cu Kα radiation source (λ=1.5406 Å) were used to record the X-ray diffraction (XRD) patterns of samples. The concentrations of Pd, P, Mo, W,

S and Pt were measured by ICP-OES (PerkinElmer, Optima 8000DV). X-ray photoelectron spectroscopy (XPS) measurements were carried out on a VG ESCALAB 220I-XL system. C 1s peak (284.8 eV) was used to calibrate the binding energy of other elements. Raman measurements were conducted on a Renishaw inViaTM conformal Raman microscope with an excitation wavelength of 532 nm and a low power density of < 5 mW/um^2. The X-ray absorption spectroscopy (XAS) data at the Pd K-edge, Mo K-edge and W L_3-edge of the samples were recorded at room temperature in the transmission mode, and the XAS data at the Pt L_3-edge were recorded at room temperature in the fluorescent mode with the 4-element silicon drift detector (SDD), at the beam line BL20U1 of the SSRF, China. The station was operated with a Si(111) double crystal monochromator. During the measurement, the storage ring was operated at an energy of 3.5 GeV and a current of 200 mA under the top-up mode. Demeter software package was used for the data processing and fitting of all the measured data.

Electrochemical measurements

Preparation of electrodes. After centrifugation of the ethanolic solution containing 1 mg of *a*-PdP@1T′-MoS_2 NPs at 8,000 rpm for 5 min, it was re-dispersed in a mixture of isopropanol (350 μL) and Milli-Q water (140 μL). Subsequently, 10 μL of Nafion solution (5 wt%, Sigma-Aldrich) was added, and the mixture was sonicated for 30 min to form a homogenous catalyst suspension. The working electrode was prepared by drop-casting 500 μL of the as-prepared catalyst suspension containing 1 mg of *a*-PdP@1T′-MoS_2 NPs on a 0.5×0.5 cm^2 carbon paper electrode (the mass loading of catalyst is 4 mg cm^{-2}). The obtained electrode was dried under ambient conditions until the solvent was completely evaporated.

Electrochemical hydrogen evolution reaction (HER) measurements. All electrochemical measurements were performed on an electrochemical workstation (CHI750e, Shanghai Chenhua Instrument Corporation, China) at ambient conditions. For the acidic HER measurements, a three-electrode system was used. The carbon paper electrode coated with the catalyst, the graphite rod and the Ag/AgCl (saturated KCl) were used as the working electrode, counter electrode and reference electrode,

respectively. All potentials were measured against the Ag/AgCl and converted to the RHE reference scale by the following equation:

$$E_{(V\ vs.\ RHE)} = E_{(V\ vs.\ Ag/AgCl)} + 0.0591 \times pH + 0.198.$$

All electrochemical measurements on three independent batches of experiments were conducted in N_2-saturated 0.5 M H_2SO_4 aqueous solution. Before the electrocatalytic measurement, the cyclic voltammetry (CV) was carried out at a potential range of 0.05−0.8 V (*vs.* RHE) to remove the capping agents on the surface of catalysts. The linear sweep voltammetric (LSV) curves were measured at ambient conditions with a scan rate of 5 mV s^{-1} between −0.4 and 0.05 V (*vs.* RHE) and then *i*R corrected based on the electrochemical impedance spectroscopy (EIS) data. Current densities were normalized by the geometric area (0.5×0.5 cm^2) of the electrode.

The electrochemically active surface area (ECSA) was determined by the following equation:

$$ECSA = \frac{C_{dl} \times S}{C_s},$$

where C_{dl} and C_s are the double-layer capacitance and specific capacitance of the sample, respectively, and *S* is the geometric surface area of the electrode (0.25 cm^2). Here, a general specific capacitance of C_s=0.04 mF cm^{-2} was used based on the previous reports.[6,7] C_{dl} was determined by the equation, $C_{dl} = i_c/v$, where i_c and v are the charging current and the scan rate, respectively. A series of CV measurements in the non-faradaic potential region of 0.25−0.35 V and 0.90−1.00 V (*vs.* RHE) under different scan rates (10, 20, 30, 40, 50, 60, 70, 80, 90, and 100 mV s^{-1}) were performed for the measurement of ECSA for HER. By plotting the measured i_c versus v, C_{dl} was obtained from the slope of the linear fitting. EIS measurements were carried out in the frequency range of 0.1 Hz−100 kHz with 10 mV amplitude to obtain the solution resistance (Rs).

Computational details.

Optimization of *a*-PdP structure. To construct the *a*-PdP structure, we performed geometry optimization based on density functional theory (DFT) and *ab initio*

molecular dynamics (AIMD) simulations using the Vienna Ab initio Simulation Package (VASP)[4,5]. We first constructed the crystalline PdP structure. Then, considering the amorphous characteristic of PdP, we conducted AIMD annealing simulations in the NVT ensemble from 2,500 to 1,000 K. The total simulation time was 4 ps with a time step of 1 fs, employing a Nosé-Hoover thermostat and a plane-wave energy cutoff of 250 eV for the electronic structure calculations. Subsequently, we performed geometry optimization on the final frame of the AIMD simulation, and the optimization was carried out using density functional theory with the generalized gradient approximation-Perdew, Burke and Ernzerhof (GGA-PBE) functional, a plane-wave cutoff energy of 300 eV, and high-precision settings. The projector augmented wave method was used to describe the core-valence interaction.[6] Ionic relaxation was performed using the conjugate gradient algorithm, with convergence thresholds set to 0.01 eV/Å for interatomic forces and 1×10^{-5} eV for energy. The optimization included both shape and volume adjustments of the crystal cell, and the D3BJ dispersion correction was incorporated to better account for van der Waals interactions.[7,8] A vacuum layer thicker than 12 Å was used to separate periodic images.

Investigation of interfacial interaction between MoS_2 and *a*-PdP. The optimized *a*-PdP structure was combined with the optimized MoS_2 structure to construct *a*-PdP@1T′-MoS_2 and *a*-PdP@1H-MoS_2 (1H-MoS_2 means the monolayer of 2H-MoS_2) heterostructures. Further AIMD simulations in the NVT ensemble at 1,000 K were performed on these heterostructures, following the same calculated details for the *a*-PdP AIMD simulation. Finally, geometry optimization was conducted on the final frame of AIMD simulation for the heterostructures, using the same parameters for the *a*-PdP optimization. Meanwhile, due to the large size of the simulated systems and to maintain consistency in calculations, only the Gamma point[9] was used for *K*-point sampling in all structures. The Lobster package was used to realize the COHP analysis.[10]

The charge density difference is defined as $\Delta\rho = \rho_{whole} - \rho_{PdP} - \rho_{MoS2}$, where ρ_{whole}, ρ_{PdP} and ρ_{MoS2} are the charge densities of the whole system, the isolated *a*-

PdP and the isolated MoS_2 ML, respectively. The iso-value of 8×10^{-3} e/Å^3 was used when the charge density differences were plotted. The binding energy is defined as $\Delta E = E_{whole} - E_{PdP} - E_{MoS2}$, where E_{whole} , E_{PdP} and E_{MoS2} are the DFT-calculated energies of the whole system, the isolated PdP and the isolated MoS_2 ML, respectively. For the structural optimization of 1T′-MoS_2 on a-PdP, since 1T′ phase is unstable at the DFT level, we first optimized the 1H-MoS_2 on a-PdP and then distorted the top-layer S atoms to build the model of 1T′-MoS_2 on a-PdP. After that, top-layer S atoms were further relaxed, while all other atoms were fixed.

Free energy calculation for HER. The adsorption free energy for hydrogen (ΔG_H) was adopted to theoretically evaluate the catalytic performance for HER, which was calculated using the following equation:[11]

$$\Delta G_H = \Delta E + \Delta E_{ZPE} - T\Delta S$$

where ΔE is the adsorption energy of H atom on the catalyst surface from DFT calculations, ΔE_{ZPE} is the correction of zero-point energy, ΔS represents the difference in entropies between the adsorbed state and the corresponding freestanding state, and T is the absolute temperature (298.15K). Free energy correction for H_2 molecule was obtained from the ideal gas approximation at 101,325 Pa.

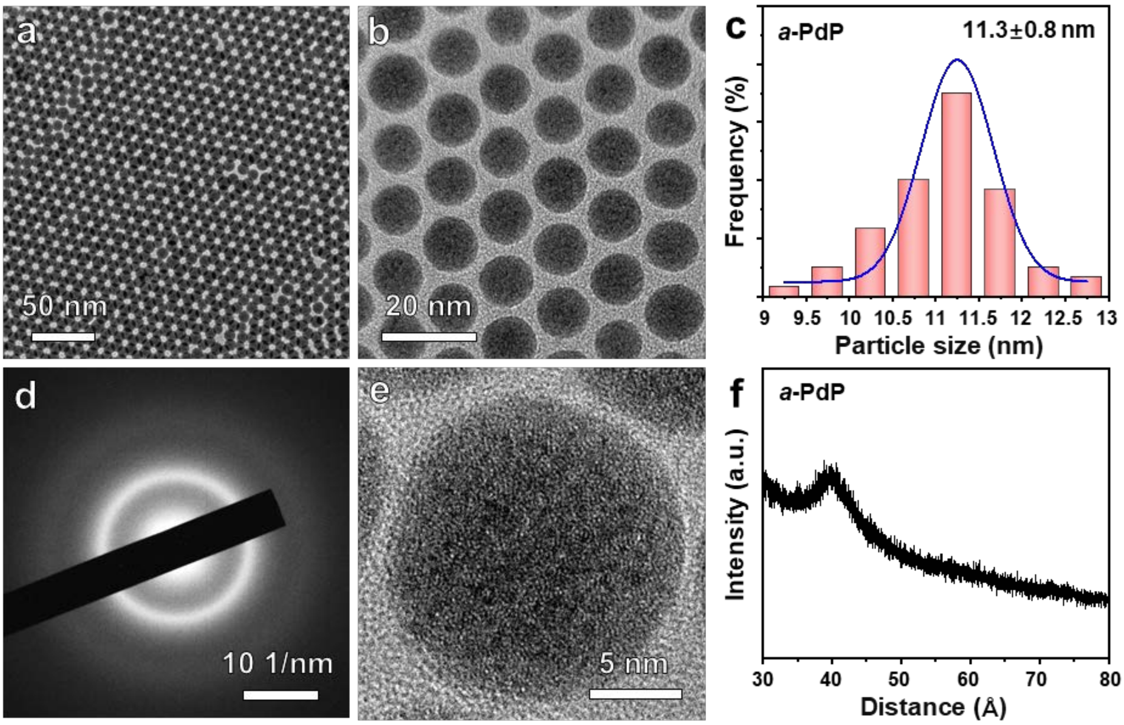

Figure S1. Characterization of *a*-PdP NPs. (a,b) Low-magnification TEM image (a), HRTEM image (b) of *a*-PdP NPs. (c) The corresponding size distribution histogram of (b). (d) SAED pattern of *a*-PdP NPs. (e) HRTEM image of a representative *a*-PdP NP, showing the amorphous structure. (f) XRD pattern of *a*-PdP NPs.

Low-magnification TEM image (Figure S1a) and HRTEM image (Figure S1b) show the sphere-like morphology of the as-prepared *a*-PdP with a size distribution of 11.3±0.8 nm (Figure S1c). The selected area electron diffraction (SAED) pattern (Figure S1d) and the HRTEM image (Figure S1e) indicate that the as-prepared *a*-PdP NPs possess an amorphous structure. As shown in Figure S1f, XRD pattern shows a broader main peak with obviously decreased intensity and disappearance of the diffraction peaks at high angles, indicating the amorphous characteristic of the synthesized *a*-PdP NPs.[12]

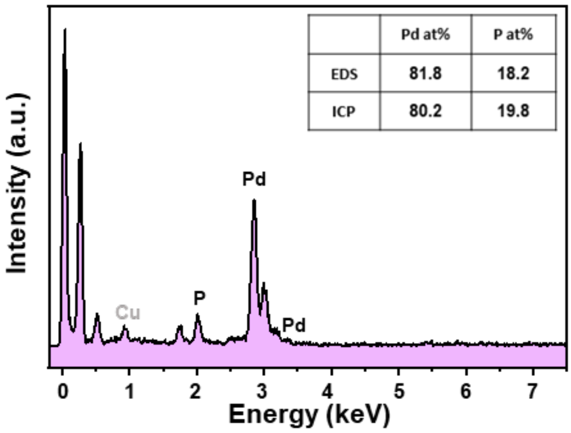

Figure S2. STEM-EDS spectrum of *a*-PdP NPs. The additional Cu signal arises from the Cu grid. Inset: atomic ratios of Pd/P obtained based on EDS and ICP-OES, showing a good agreement.

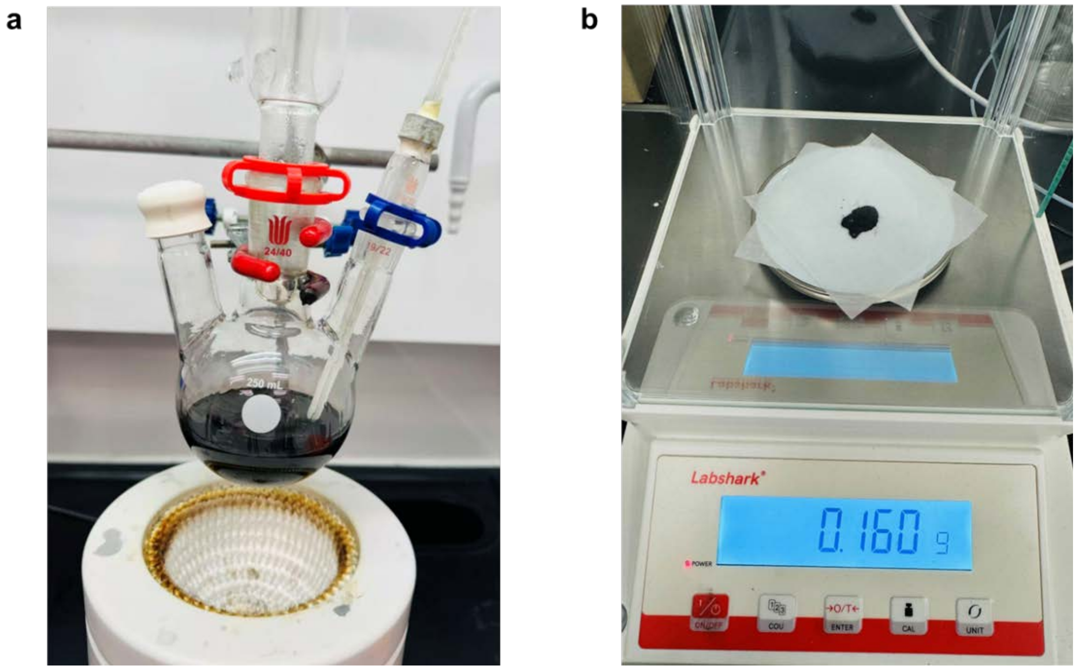

Figure S3. Scale-up synthesis of *a*-PdP@1T′-MoS_2 NPs in a single batch of experiment. (a) Photograph of the *a*-PdP@1T′-MoS_2 NPs synthesized in a 250-mL three-necked flask. (b) The weight of *a*-PdP@1T′-MoS_2 NPs (~160 mg) synthesized in a single batch of experiment.

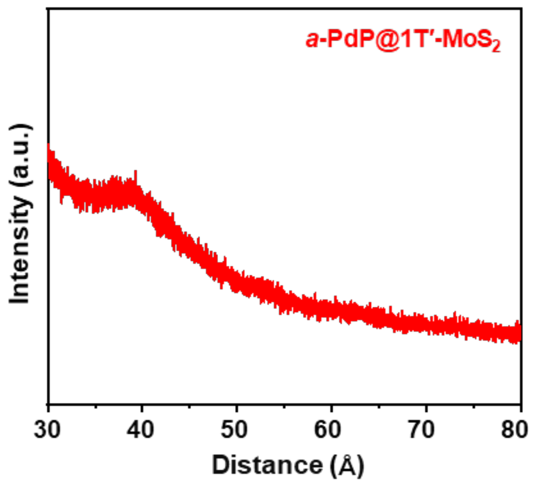

Figure S4. XRD pattern of *a*-PdP@1T′-MoS_2 NPs.

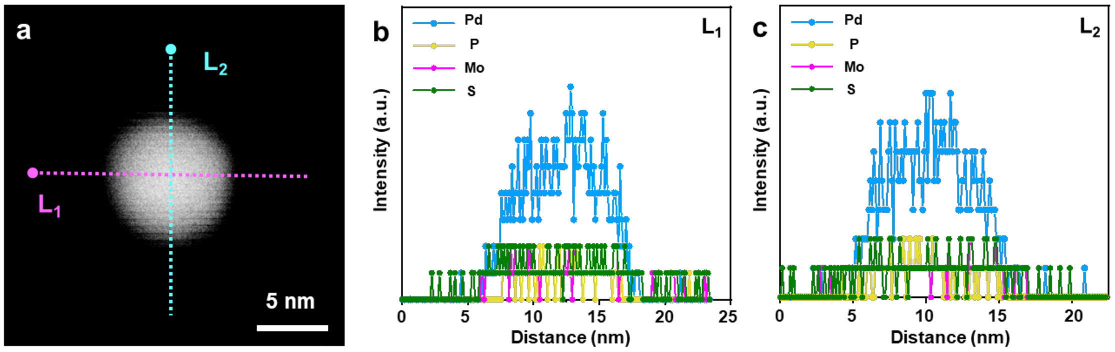

Figure S5. STEM-EDS characterization of a representative *a*-PdP@1T′-MoS_2 NP. (a-c) STEM image of an *a*-PdP@1T′-MoS_2 NP (a) and the corresponding STEM-EDS elemental line scans along the pink dotted line (b) and the blue dotted line (c) in (a).

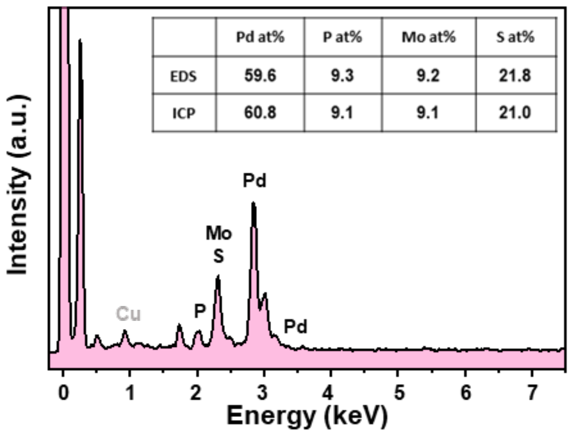

Figure S6. STEM-EDS spectrum of *a*-PdP@1T′-MoS_2 NPs. The additional Cu signal arises from the Cu grid. Inset: atomic ratios of Pd/P/Mo/S obtained based on EDS and ICP-OES, showing a good agreement.

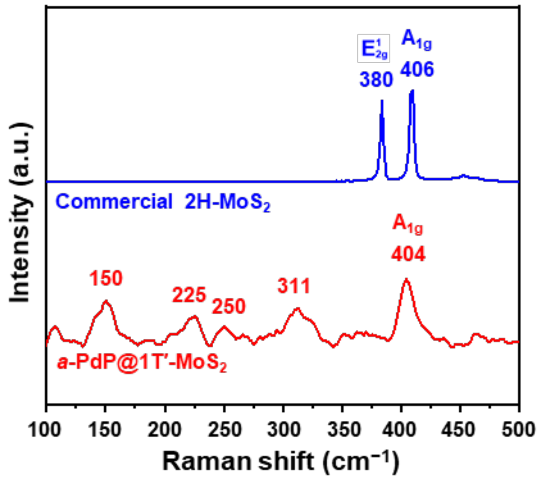

Figure S7. Raman spectra of the as-prepared *a*-PdP@1T′-MoS_2 NPs and commercial 2H-MoS_2.

Table S1. EXAFS fitting parameters at the Mo K edge for various samples*.

Sample	Shell	CN^a	R(Å)^b	σ^2(Å^2)^c	ΔE_0(eV)^d	R factor
2H-MoS_2	Mo−S	6.0	2.41	0.003	1.6	0.006
	Mo−Mo	6.0	3.17	0.003		
a-PdP@1T′-MoS_2	Mo−S	4.5	2.41	0.005	−0.1	0.015
	Mo−Mo	1.4	2.77	0.008		
a-PdP@1T′-$MoWS_2$	Mo−S	4.3	2.40	0.005	1.2	0.016
	Mo−Mo/W	0.8	2.81	0.005		

[a]*CN*, coordination number; [b]*R*, the distance to the neighboring atom; [c]σ^2, the mean square relative displacement (MSRD); [d]ΔE_0, inner potential correction; *R* factor indicates the goodness of the fit. *This value was fixed during EXAFS fitting, based on the known structure of Mo. Fitting range: 3.0 ≤ k (/Å) ≤ 11.4 and 1.0 ≤ R (Å) ≤ 2.9 (*a*-PdP@1T′-MoS_2), 3.0 ≤ k (/Å) ≤ 12.5 and 1.5 ≤ R (Å) ≤ 3.0 (*a*-PdP@1T′-$MoWS_2$), 3.0 ≤ k (/Å) ≤ 12.9 and 1.2 ≤ R (Å) ≤ 3.2 (2H-MoS_2).

Table S2. EXAFS fitting parameters at the Pd *K* edge for various samples*.

Sample	Shell	CN^a	R(Å)^b	σ^2(Å^2)^c	ΔE_0(eV)^d	R factor
fcc-Pd foil	Pd−Pd	12.0	2.74	0.005	4.3	0.003
PdS	Pd−S	6.0	2.31	0.007	5.4	0.005
a-PdP	Pd−P	1.2	2.27	0.013	−3.9	0.001
	Pd−Pd	6.5	2.76	0.003		
a-PdP@1T′-MoS_2	Pd−P	0.6	2.28	0.003	6.1	0.004
	Pd−S	1.5	2.38	0.007		
	Pd−Pd	4.2	2.75	0.012	−3.2	

[a]*CN*, coordination number; [b]*R*, the distance to the neighboring atom; [c]σ^2, the mean square relative displacement (MSRD); [d]ΔE_0, inner potential correction; *R* factor indicates the goodness of the fit. *This value was fixed during EXAFS fitting, based on the known structure of Pd. Fitting range: 3.0 ≤ k (/Å) ≤ 14.7 and 1.5 ≤ R (Å) ≤ 3.0 (*fcc*-Pd foil), 2.2 ≤ k (/Å) ≤ 12.7 and 1.0 ≤ R (Å) ≤ 2.3 (PdS), 3.0 ≤ k (/Å) ≤ 11.0 and 1.1 ≤ R (Å) ≤ 3.0 (*a*-PdP@1T′-MoS_2).

Note S1

By using the synthesized *a*-PdP NPs (Figure S1) as templates, 1T′-WS_2 MLs can be grown on the *a*-PdP NPs to form *a*-PdP@1T′-WS_2 core-shell NPs *via* a facile and rapid wet-chemical route (see Supporting Information for details). As shown in the low-magnification TEM image (Figure 3a), the as-prepared *a*-PdP@1T′-WS_2 maintains the sphere-like morphology. The SAED pattern (Figure 3b) of *a*-PdP@1T′-WS_2 NPs confirms the PdP NPs still maintain amorphous after the growth of 1T′-WS_2 ML, which is further confirmed by the XRD pattern (Figure S8), suggesting a good structural stability of *a*-PdP. The HAADF-STEM images (Figure 3c,d) reveal that a WS_2 ML, composed of a layer of W atoms which is sandwiched between two layers of S atoms, is uniformly grown on the *a*-PdP NP. The STEM-EDS elemental maps (Figure 3e) and STEM-EDS line scanning profiles (Figure S9) corroborate the even distribution of Pd, P, W and S in the synthesized *a*-PdP@1T′-WS_2 NPs. Based on the STEM-EDS result, the Pd/P/W/S atomic ratio of the obtained *a*-PdP@1T′-WS_2 NPs is determined to be 55.3/9.9/11.7/23.9, which is in accordance with the ICP-OES result (Figure S10).

Furthermore, Raman spectroscopy, XPS, XANES and EXAFS were used to characterize the synthesized 1T′-WS_2 MLs with high phase purity on *a*-PdP NPs. For comparison, the commercial 2H-WS_2 was also characterized. As shown in the Raman spectrum of the *a*-PdP@1T′-WS_2 NPs (red curve in Figure S11a), five distinctive peaks located at 128, 204, 260, 312 and 408 cm^{-1} (A_{1g}), respectively, are observed, which are similar to the reported single-layer 1T′-WS_2.[13] The characteristic Raman peak of 2H-WS_2 located at 350 cm^{-1} (E^1_{2g}) (blue curve in Figure S11a) has not been observed in the *a*-PdP@1T′-WS_2 NPs, indicating the high phase purity of synthesized 1T′-WS_2 MLs. XPS spectrum of *a*-PdP@1T′-WS_2 NPs (red curve in Figure S11b) shows two characteristic peaks located at 33.8 and 31.7 eV, which can be assigned to the W $4f_{5/2}$ and W $4f_{7/2}$ of 1T′-WS_2, respectively. Both of them shift to a lower binding energy in comparison with the W $4f_{5/2}$ (34.6 eV) and W $4f_{7/2}$ (32.4 eV) of 2H-WS_2 (blue curve in Figure S11b), which is consistent with the previous reports.[13,14] Moreover, XANES and EXAFS measurements were conducted to investigate the electronic structure and coordination environment of the as-synthesized 1T′-WS_2. The W L_3-edge XANES spectrum of 1T′-WS_2 MLs on *a*-PdP NPs (red curve in Figure S11c) displays different characteristics as

compared to the commercial 2H-WS_2 (blue curve in Figure S11c) due to the difference phases.[14] The local coordination environment of W in the WS_2 was investigated by the Fourier transform (FT) of EXAFS spectra in R space (Figure S11d) and the fitting results (Table S3). Compared to the W−W bond distance of 3.21 Å in 2H-WS_2, a significantly shorter W−W bond distance of 2.75 Å is observed in the 1T′-WS_2 MLs on *a*-PdP NPs (Table S3), which could arise from the structural distortion characteristic of the 1T′ phase.[13]

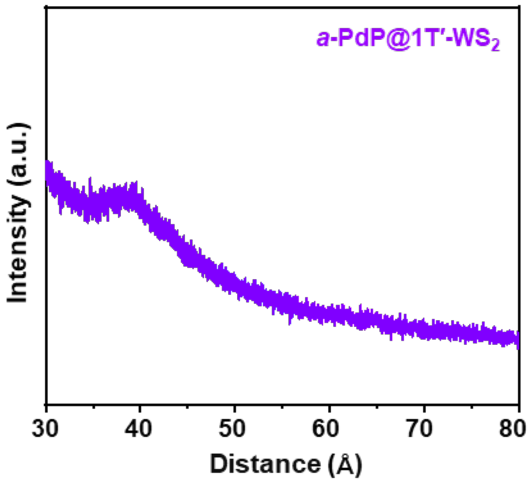

Figure S8. XRD pattern of *a*-PdP@1T′-WS_2 NPs.

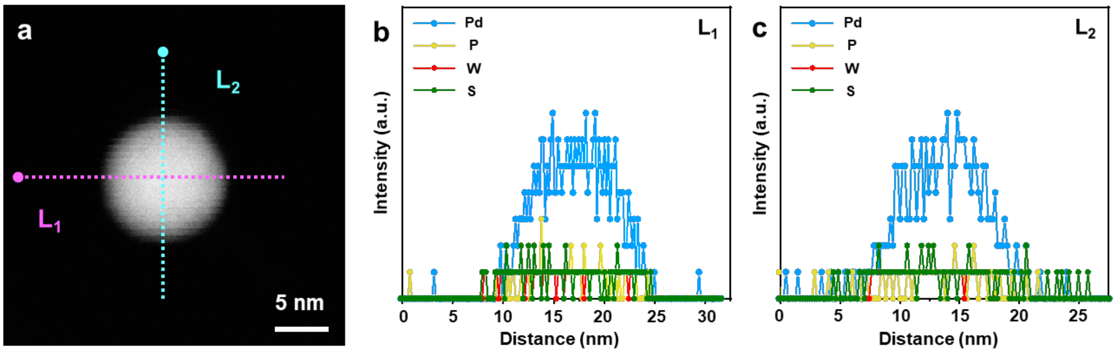

Figure S9. STEM-EDS characterization of a representative *a*-PdP@1T′-WS_2 NP. (a-c) STEM image of an *a*-PdP@1T′-WS_2 NP (a) and the corresponding STEM-EDS elemental line scans along the pink dotted line (b) and the blue dotted line (c) in (a).

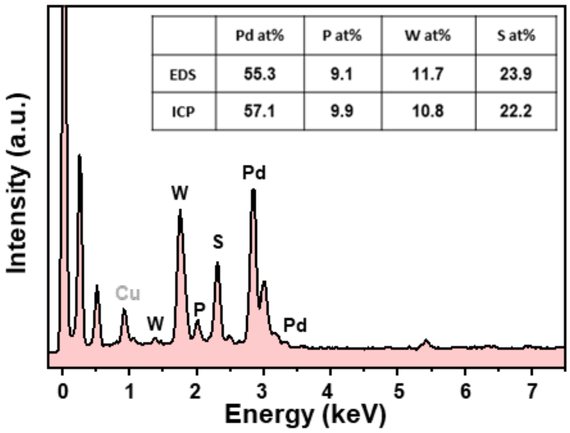

Figure S10. STEM-EDS spectrum of *a*-PdP@1T′-WS_2 NPs. The additional Cu signal arises from the Cu grid. Inset: atomic ratios of Pd/P/W/S obtained based on EDS and ICP-OES, showing a good agreement.

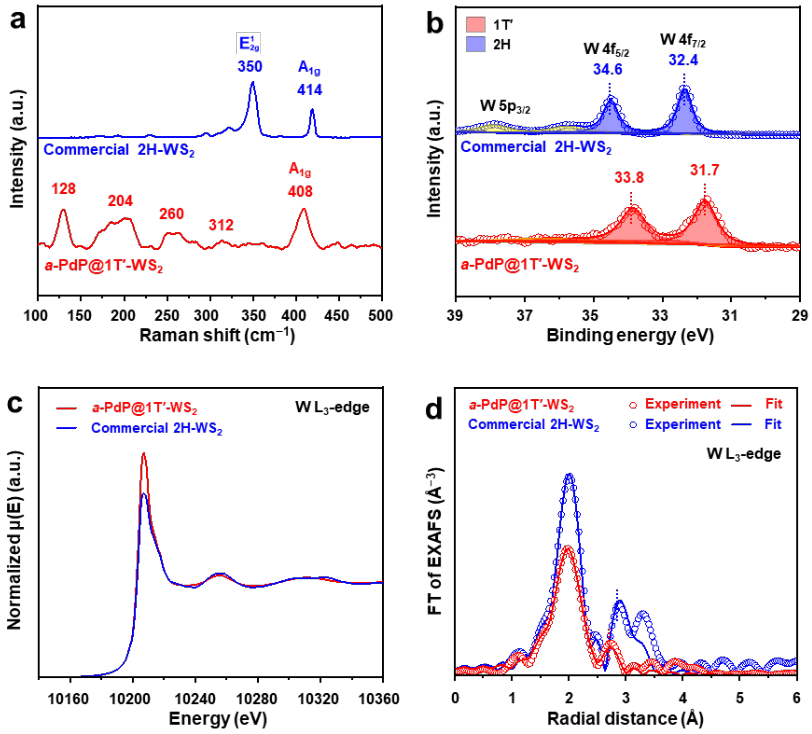

Figure S11. Characterization of the as-prepared *a*-PdP@1T′-WS_2 NPs. (a-d) Raman spectra (a), high-resolution XPS W 4f spectra (b), W L_3-edge XANES spectra (c), and Fourier transformed W L_3-edge EXAFS spectra in R space (d) of *a*-PdP@1T′-WS_2 NPs and commercial 2H-WS_2.

Table S3. EXAFS fitting parameters at the W L_3 edge for various samples*.

Sample	Shell	CN^a	R(Å)^b	σ^2(Å^2)^c	ΔE_0(eV)^d	R factor
2H-WS_2	W−S	6.0	2.40	0.003	7.1	0.007
	W−W	6.0	3.21	0.004		
a-PdP@1T′-WS_2	W−S	5.6	2.41	0.007	4.8	0.003
	W−W	2.2	2.75	0.006		
a-PdP@1T′-$MoWS_2$	W−S	4.8	2.39	0.006	4.3	0.011
	W−W/Mo	1.5	2.71	0.008		

[a]*CN*, coordination number; [b]*R*, the distance to the neighboring atom; [c]σ^2, the mean square relative displacement (MSRD); [d]ΔE_0, inner potential correction; *R* factor indicates the goodness of the fit. *This value was fixed during EXAFS fitting, based on the known structure of W. Fitting range: $3.0 \le k$ (/Å) ≤ 12.5 and $1.0 \le R$ (Å) ≤ 3.0 (*a*-PdP@1T′-WS_2), $3.0 \le k$ (/Å) ≤ 12.5 and $1.0 \le R$ (Å) ≤ 3.0 (*a*-PdP@1T′-$MoWS_2$), $3.0 \le k$ (/Å) ≤ 12.8 and $1.2 \le R$ (Å) ≤ 3.2 (2H-WS_2).

Note S2

By using the synthesized *a*-PdP NPs (Figure S1) as templates, 1T′-$MoWS_2$ MLs can be grown on the *a*-PdP NPs to form *a*-PdP@1T′-$MoWS_2$ core-shell NPs *via* a facile and rapid wet-chemical route (see Supporting Information for details). As shown in the low-magnification TEM image (Figure 3f), the as-prepared *a*-PdP@1T′-$MoWS_2$ maintains the sphere-like morphology. The SAED pattern (Figure 3g) of *a*-PdP@1T′-$MoWS_2$ NPs confirms the PdP NPs still maintain amorphous after the growth of 1T′-$MoWS_2$ ML, which is further confirmed by the XRD pattern (Figure S12), suggesting a good structural stability of *a*-PdP. The HAADF-STEM images (Figure 3h,i) reveal that a $MoWS_2$ ML, composed of a layer of Mo/W atoms which is sandwiched between two layers of S atoms, is uniformly grown on the *a*-PdP NP. The STEM-EDS elemental maps (Figure 3j) and STEM-EDS line scanning profiles (Figure S13) corroborate the even distribution of Pd, P, Mo, W and S in the synthesized *a*-PdP@1T′-$MoWS_2$ NPs. Based on the STEM-EDS result, the Pd/P/Mo/W/S atomic ratio of the obtained *a*-PdP@1T′-WS_2 NPs is determined to be 60.8/8.9/3.0/7.0/20.3, which is in accordance with the corresponding ICP-OES result (Figure S14).

Furthermore, XPS, XANES and EXAFS were used to characterize the as-synthesized 1T′-$MoWS_2$ MLs on *a*-PdP NPs. For comparison, the *a*-PdP@1T′-MoS_2 and *a*-PdP@1T′-WS_2 NPs as well as the commercial 2H-MoS_2 and 2H-WS_2 were also characterized. XPS spectrum of *a*-PdP@1T′-$MoWS_2$ NPs (pink curve in Figure S15a) shows two characteristic peaks located at 231.2 and 228.0 eV, which can be assigned to the Mo $3d_{3/2}$ and Mo $3d_{5/2}$ of 1T′-$MoWS_2$, respectively. Both of them shift to lower binding energy by ~0.3 eV in comparison with the Mo $3d_{3/2}$ (231.5 eV) and Mo $3d_{5/2}$ (228.3 eV) of *a*-PdP@1T′-MoS_2 (red curve in Figure S15a), and ~1.2 eV in comparison with the Mo $3d_{3/2}$ (232.4 eV) and Mo $3d_{5/2}$ (229.2 eV) of 2H-MoS_2 (blue curve in Figure S15a), which are consistent with the previous reports.[15,16] Moreover, the Mo K-edge XANES spectrum of 1T′-$MoWS_2$ MLs (pink curve in Figure S15b) on *a*-PdP NPs displays a lower chemical state compared to the commercial 2H-MoS_2 (blue curve in Figure S15b) due to the more electron-rich state on the 1T′-$MoWS_2$ surface, while a higher chemical state compared to the *a*-PdP@1T′-MoS_2 (red curve in Figure S15b) owing to the electron transfer from Mo to neighboring W atoms.[16,17] As shown in the FT of EXAFS spectra in R space (Figure

S15c) and the fitting results (Table S1), the *a*-PdP@1T′-$MoWS_2$ NPs (pink curve in Figure S15c) exhibit a Mo−Mo/W bond distance of 2.81 Å. This value is shorter than the Mo−Mo bond distance in the 2H-MoS_2 (3.17 Å, blue curve in Figure S15c), arising from the structural distortion in the 1T′ phase.[17] Meanwhile, it is longer than the Mo−Mo bond distance in the *a*-PdP@1T′-MoS_2 NPs (2.77 Å, red curve in Figure S15c), likely due to the incorporation of neighboring W atoms with larger atomic size.

Moreover, XPS spectrum of *a*-PdP@1T′-$MoWS_2$ NPs (pink curve in Figure S15d) shows two characteristic peaks located at 34.4 and 32.3 eV, which can be assigned to the W $4f_{5/2}$ and W $4f_{7/2}$ of 1T′-$MoWS_2$, respectively. Both of them shift to a higher binding energy by ~0.6 eV in comparison with the W $4f_{5/2}$ (33.8 eV) and W $4f_{7/2}$ (31.7 eV) of *a*-PdP@1T′-WS_2 (red curve in Figure S15d), and a lower binding energy in comparison with the W $4f_{5/2}$ (34.6 eV) and W $4f_{7/2}$ (32.4 eV) of 2H-WS_2 (blue curve in Figure S15d), which are consistent with the previous reports.[13,14] Furthermore, the pre-edge of W L_3-edge XANES spectrum of 1T′-$MoWS_2$ MLs (pink curve in Figure S15e) on *a*-PdP NPs is different from the *a*-PdP@1T′-MoS_2 (red curve in Figure S15e) and commercial 2H-MoS_2 (blue curve in Figure S15e) due to the different structural characteristics.[13] As shown in the FT of EXAFS spectra in R space (Figure S15f) and the fitting results (Table S3), the *a*-PdP@1T′-$MoWS_2$ NPs (pink curve in Figure S15f) exhibit a W−W/Mo bond distance of 2.71 Å. This value is shorter than the W−W bond distance in the 2H-WS_2 (3.21 Å, blue curve in Figure S15f), arising from the structural distortion in the 1T′ phase.[13,14] Meanwhile, it is shorter than the W−W bond distance in the *a*-PdP@1T′-WS_2 NPs (2.75 Å, red curve in Figure S15f), likely due to the incorporation of neighboring Mo atoms with smaller atomic size.

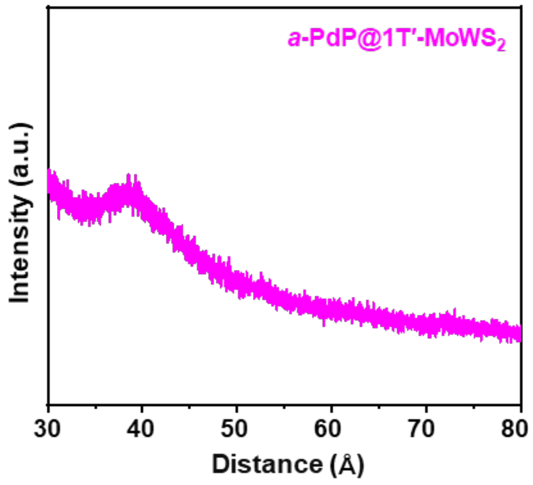

Figure S12. XRD pattern of *a*-PdP@1T′-$MoWS_2$ NPs.

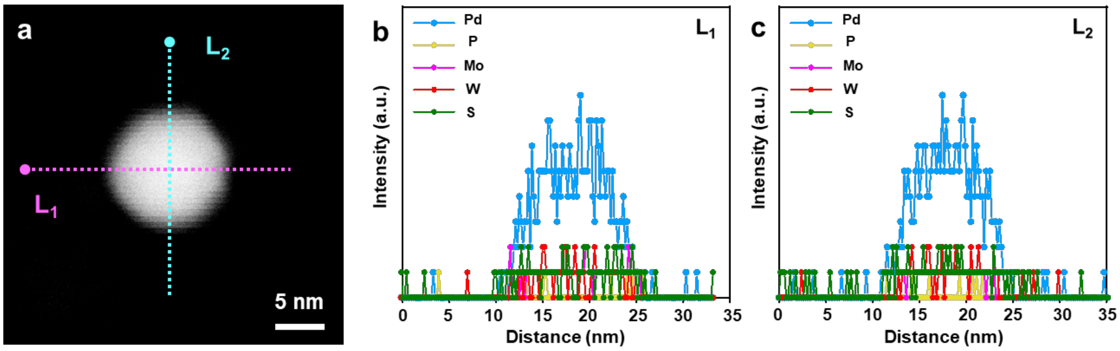

Figure S13. STEM-EDS characterization of a representative *a*-PdP@1T′-$MoWS_2$ NP. (a-c) STEM image of an *a*-PdP@1T′-$MoWS_2$ NP (a) and the corresponding STEM-EDS elemental line scans along the pink dotted line (b) and the blue dotted line (c) in (a).

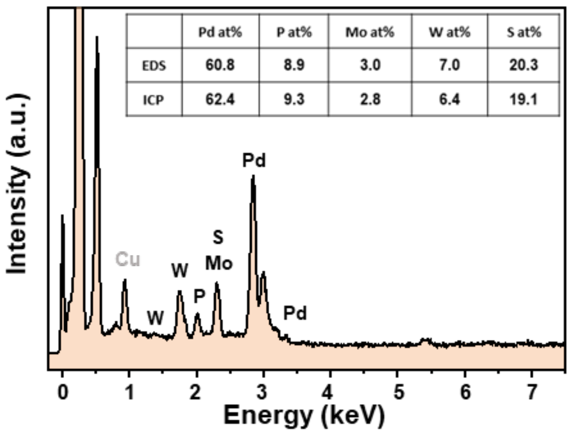

Figure S14. STEM-EDS spectrum of *a*-PdP@1T′-$MoWS_2$ NPs. The additional Cu signal arises from the Cu grid. Inset: atomic ratios of Pd/P/Mo/W/S obtained based on EDS and ICP-OES, showing a good agreement.

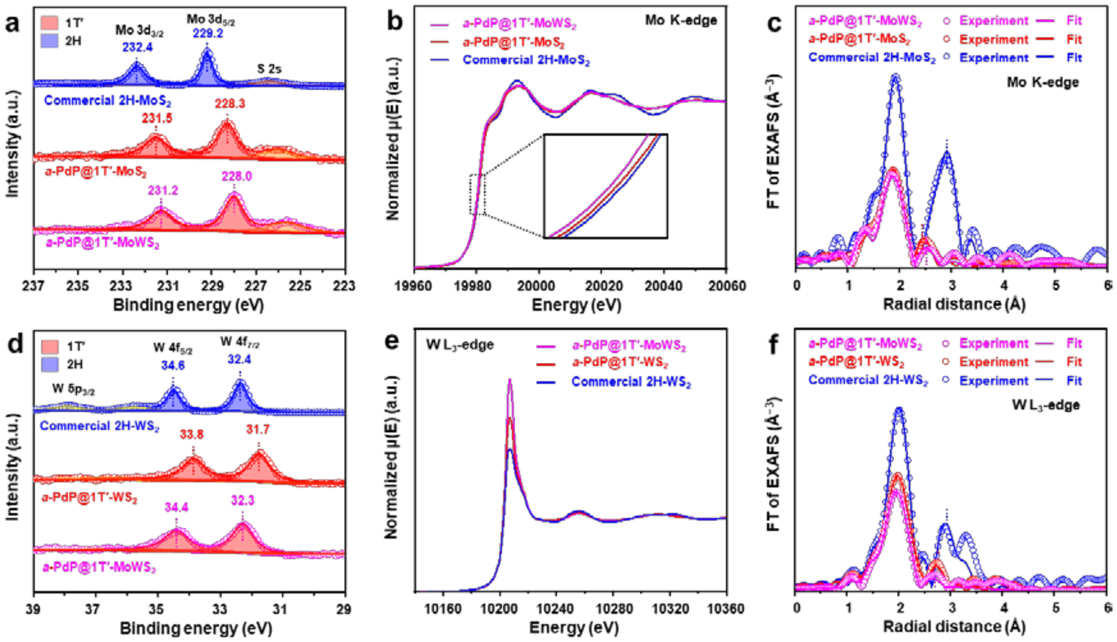

Figure S15. Characterization of the as-prepared *a*-PdP@1T′-$MoWS_2$ NPs. (a-c) High-resolution XPS Mo 3d spectra (a), Mo K-edge XANES spectra (b) and Fourier transformed Mo K-edge EXAFS spectra in R space (c) of the as-prepared *a*-PdP@1T′-$MoWS_2$ NPs, *a*-PdP@1T′-MoS_2 NPs and commercial 2H-MoS_2. (d-f) High-resolution XPS W 4f spectra (d), W L_3-edge XANES spectra (e) and Fourier transformed W L_3-edge EXAFS spectra in R space (f) of the as-prepared *a*-PdP@1T′-$MoWS_2$ NPs, *a*-PdP@1T′-WS_2 NPs and commercial 2H-WS_2.

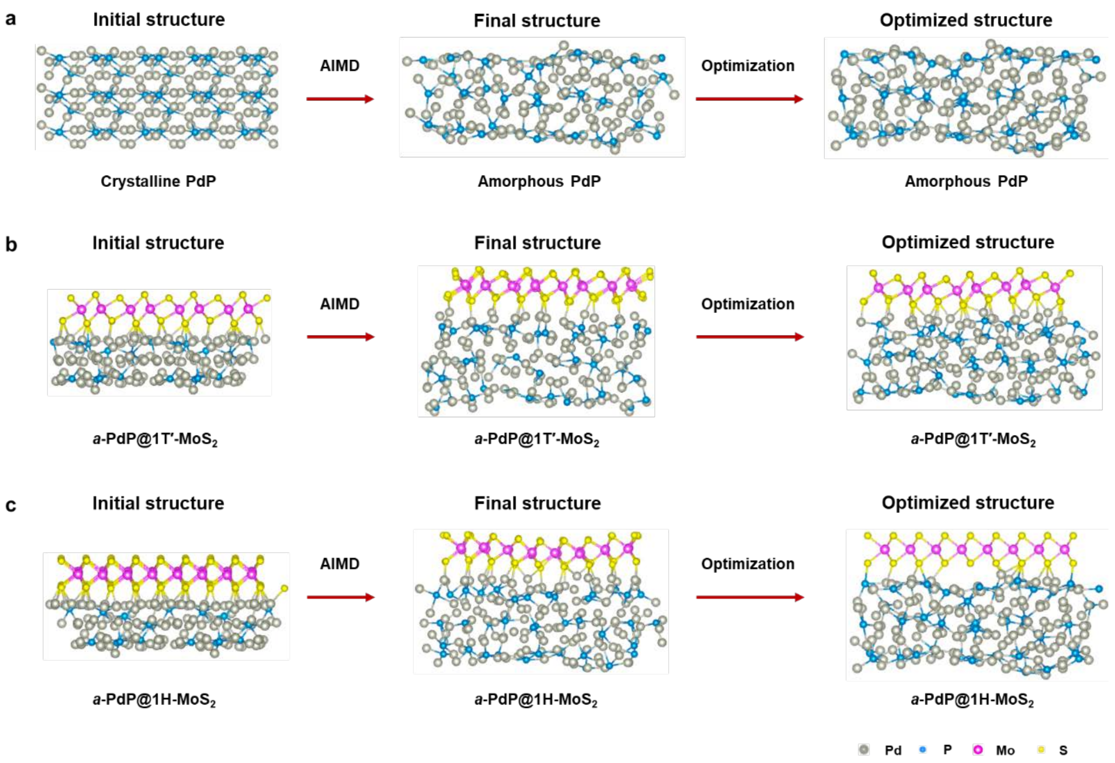

Figure S16. (a-c) Schematic illustrations of the optimization for *a*-PdP (a), *a*-PdP@1T′-MoS_2 (b) and *a*-PdP@1H-MoS_2 (c) by AIMD. The grey, blue, pink and golden balls represent Pd, P, Mo and S atoms, respectively.

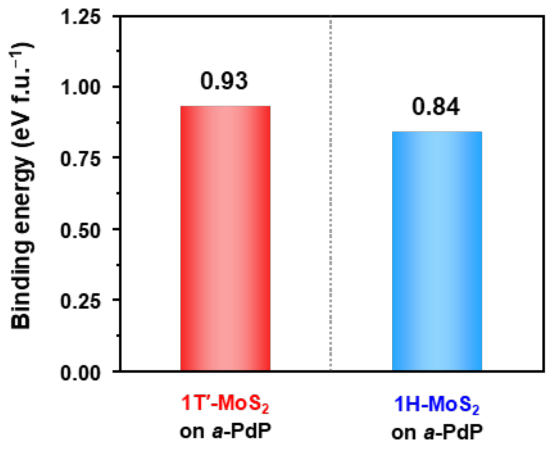

Figure S17. Calculated binding energies between 1T′-MoS_2 ML and *a*-PdP, and between 1H-MoS_2 and *a*-PdP.

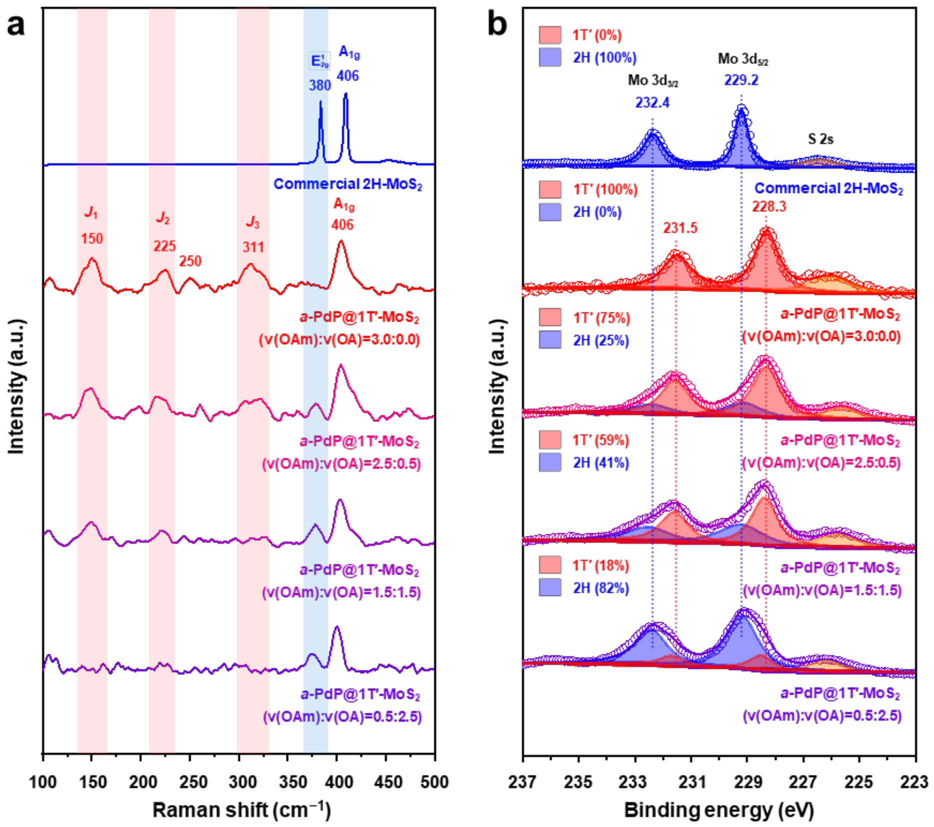

Figure S18. Characterization of the phase purity of MoS_2 MLs grown on *a*-PdP NPs with varying oleylamine concentrations. (a) Raman spectra and (b) high-resolution XPS Mo 3d spectra of *a*-PdP@MoS_2 NPs synthesized in a mixed solution of oleylamine (OAm) and oleic acid (OA) with their volume ratios of 3.0:0.0, 2.5:0.5, 1.5:1.5 and 0.5:2.5, compared with the commercial 2H-MoS_2.

To further confirm the role of oleylamine in synthesizing and stabilizing the 1T′-MoS_2 MLs on *a*-PdP NPs, additional control experiments were conducted, *i.e.*, oleylamine was mixed with oleic acid during the synthesis of *a*-PdP@MoS_2 NPs. As shown in the Raman spectra (Figure S18a), when the volume ratio of oleylamine and oleic acid is successively reduced from 3.0:0.0 to 2.5:0.5, 1.5:1.5, and 0.5:2.5, the intensities of the three characteristic Raman peaks, located at 150 (J_1), 225 (J_2) and 311 cm^{-1} (J_3), respectively,[17] of 1T′-MoS_2 MLs, gradually decrease, while the intensity of the characteristic 2H-MoS_2 peak at 380 cm^{-1} (E^1_{2g})[17] gradually increases. As shown in Figure S18b, the XPS spectra of Mo 3d peaks can be deconvoluted into 1T′-MoS_2 (light red) and 2H-MoS_2 (light blue). Based on the fitting of these peaks, the percentages of 1T′ and 2H phases could be quantified. As a result, with the successive decrease in the volume ratio

of oleylamine and oleic acid, the phase purity of 1T′-MoS_2 MLs gradually decreases from 100% to 75%, 59%, and 18%. These results further confirm the crucial role of oleylamine in the growth and stabilization of 1T′-MoS_2 MLs on *a*-PdP NPs.

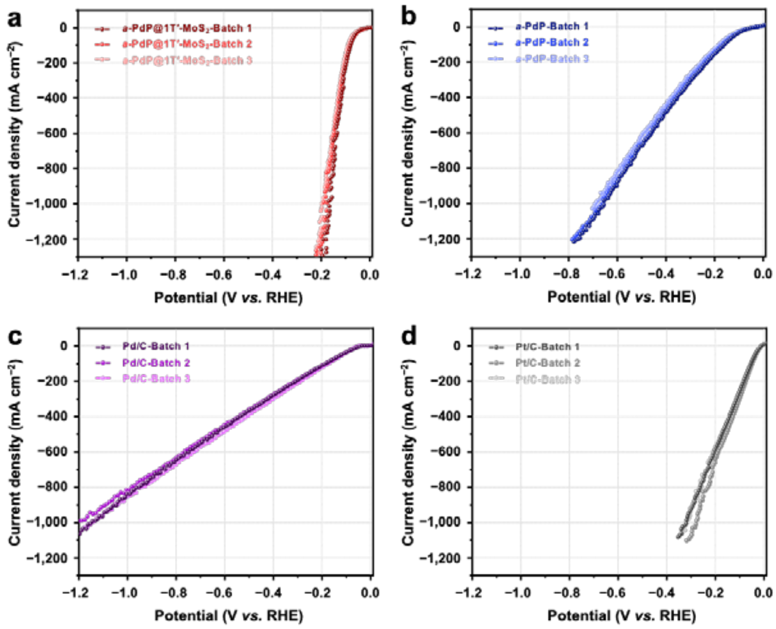

Figure S19. Electrocatalytic HER performance of *a*-PdP@1T′-MoS_2 NPs and other catalysts on three independent batches of experiments. (a-d) HER polarization curves of *a*-PdP@1T′-MoS_2 (a), *a*-PdP (b), commercial Pd/C (c), and commercial Pt/C (d) recorded in N_2-saturated 0.5 M H_2SO_4 electrolyte at a scan rate of 5 mV s^{-1} on three independent batches of experiments.

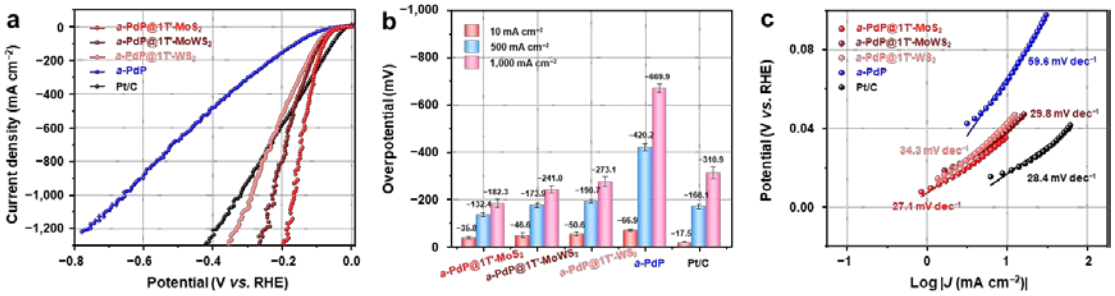

Figure S20. Electrocatalytic HER performance of *a*-PdP@1T′-TMD NPs. (a) HER polarization curves of *a*-PdP@1T′-MoS_2, *a*-PdP@1T′-$MoWS_2$, *a*-PdP@1T′-WS_2, *a*-PdP NPs, and commercial Pt/C recorded in N_2-saturated 0.5 M H_2SO_4 electrolyte at a scan rate of 5 mV s^{-1}. (b) Comparison of the overpotentials for different HER catalysts at the current densities of 10, 500 and 1,000 mA cm^{-2}, respectively. The error bar represents the standard deviation (SD). (c) Tafel plots for HER obtained from the corresponding polarization curves in (a).

As shown in Figure S20a,b, the *a*-PdP@1T′-MoS_2 catalyst achieves current densities of 10, 500 and 1,000 mA cm^{-2} at low overpotentials of only −36.2, −126.2 and −165.8 mV, respectively, which are lower than that of *a*-PdP@1T′-$MoWS_2$ NPs (−39.3, −168.6 and −229.0 mV, respectively), *a*-PdP@1T′-WS_2 NPs (−43.1, −193.0 and −290.0 mV, respectively) and *a*-PdP NPs (−64.1, −408.8 and −653.2 mV, respectively), demonstrating its superior HER activity. As shown in Figure S20c, the Tafel slope of *a*-PdP@1T′-MoS_2 is 27.1 mV dec^{-1}, which is lower than that of Pt/C (28.4 mV dec^{-1}), *a*-PdP@1T′-$MoWS_2$ (29.8 mV dec^{-1}), *a*-PdP@1T′-WS_2 (34.3 mV dec^{-1}) and *a*-PdP (59.6 mV dec^{-1}), suggesting its faster HER reaction kinetics.

Table S4. Summary of some representative MoS_2-based catalysts for acidic HER reported in the literature and this work.

Materials	Electrolytes	Overpotential [η_{10}, mV]	Tafel slope [mV dec^{-1}]	Long-term stability	Reference
***a*-PdP@1T′-MoS_2**	**0.5 M H_2SO_4**	**−35.8**	**27.1**	**>500 h@500 mA cm^{-2}**	**This work**
Co-MoS_2 NS	0.5 M H_2SO_4	−56	32	1,000 cycles	18
Pt/2H-MoS_2	0.5 M H_2SO_4	−59	50	1,000 cycles	19
MoS_2/$CoSe_2$	0.5 M H_2SO_4	−68	36	20 h@700 mV	20
1T′-MoS_2 edge	0.5 M H_2SO_4	−77	~60	N.A.	21
Pd-2H/1T-MoS_2	0.5 M H_2SO_4	−78	32	5,000 cycles	22
1T′-MoS_2 NRBs	0.5 M H_2SO_4	−79	36.2	10,000 cycles<br>20 h@80 mV	23
(N, PO_4^{3-})-MoS_2/VG	0.5 M H_2SO_4	−85	42	1,000 cycles<br>10 h@10 mA cm-2	24
MoS_2/Au	0.5 M H_2SO_4	−90	69	1,000 cycles	25
Se-MoS_2	0.5 M H_2SO_4	−104	59	24 h@10 mA cm^{-2}	26
AMF screw MoS_2	0.5 M H_2SO_4	−105	45	500 cycles	27
N-MoS_2/CN	0.5 M H_2SO_4	−114	46.8	1,000 cycles<br>10 h@155 mV	28
MoS_2 defect rich (hydrothermal)	0.5 M H_2SO_4	−120	50	3,000 cycles<br>10,000 s@200 mV	29
Au-MoS_2	0.5 M H_2SO_4	−120	71	N.A.	30
Oxygenated MoS_2	0.5 M H_2SO_4	−130	67	N.A.	31
Zn-MoS_2	0.5 M H_2SO_4	−130	51	1,000 cycles	32
Etched MoS_2 NS Arrays	0.5 M H_2SO_4	−131	48	1,000 cycles	32
Co/Se-MoS_2 nanofoam	0.5 M H_2SO_4	−132	75	30 h@10 mA cm^{-2}	34
Co-MoS_2	0.5 M H_2SO_4	−137	59	N.A.	35
SLHS-1T-MoS_2	0.5 M H_2SO_4	−137	40	10,000 cycles	36
Pd/1T-MoS_2	0.5 M H_2SO_4	−140	50	1,000 cycles	37
1T′-MoS_2 HSs	0.5 M H_2SO_4	−147	42.4	200 h@1 A cm^{-2}	38

MoS_2/RGO	0.5 M H_2SO_4	−150	41	1,000 cycles	39
Porous 1T′-MoS_2	0.5 M H_2SO_4	−153	43	1,000 cycles<br>20,000 s@180 mV	40
Ni-Co-MoS_2 nanobox	0.5 M H_2SO_4	−155	52	1,000 cycles<br>12 h@155 mV	41
1T‴-MoS_2-V_S	0.5 M H_2SO_4	−158	74.5	24 h@158 mV	42
MCM@MoS_2-Ni	0.5 M H_2SO_4	−161	81	1,000 cycles<br>24 h@250 mV	43
FD MoS_2-ML	0.5 M H_2SO_4	−164	36	N.A.	44
Hierarchical MoS_2 NSs	0.5 M H_2SO_4	−167	70	1,000 cycles	45
Mo SAs/ML-MoS_2	0.5 M H_2SO_4	−170	40	9 cycles	46
S_V-MoS_2	0.5 M H_2SO_4	−170	60	N.A.	47
Metallic-phase MoS_2	0.5 M H_2SO_4	−175	41	1,000 cycles	48
1T′-MoS_2	0.5 M H_2SO_4	−176	100	N.A.	16
Zn SAs/1T-MoS_2	0.5 M H_2SO_4	−177	84.9	3,000 cycles	49
1T-MoS_2	0.5 M H_2SO_4	−187	43	1,000 cycles	50
Zn@MoS_2	0.5 M H_2SO_4	−194	73	1,000 cycles	51
Ag@MoS_2-11.3 nm	0.5 M H_2SO_4	−195.7	41.1	10,000 cycles	52
Ru and O co-doped MoS_2	0.5 M H_2SO_4	−197	75.8	N.A.	53
1T′-MoS_2 (CVD)	0.5 M H_2SO_4	−205	51	1,000 cycles	15
Double gyroid MoS_2	0.5 M H_2SO_4	−206	50	N.A.	54
Pt/1T-MoS_2	0.5 M H_2SO_4	−210	104	N.A.	55

Abbreviation: NS, nanosheet; NRB, nanoribbon; VG, vertical graphene; AMF, alternating magnetic field; SLHS, single-layer hollow sphere; HS, hollow structure; RGO, reduced graphene oxide; V_S, S vacancies; MCM, multichannel carbon matrix; FD, Frenkel-defected; ML, monolayer; SA, single atom; S_V, S vacancies; CVD, chemical vapor deposition.

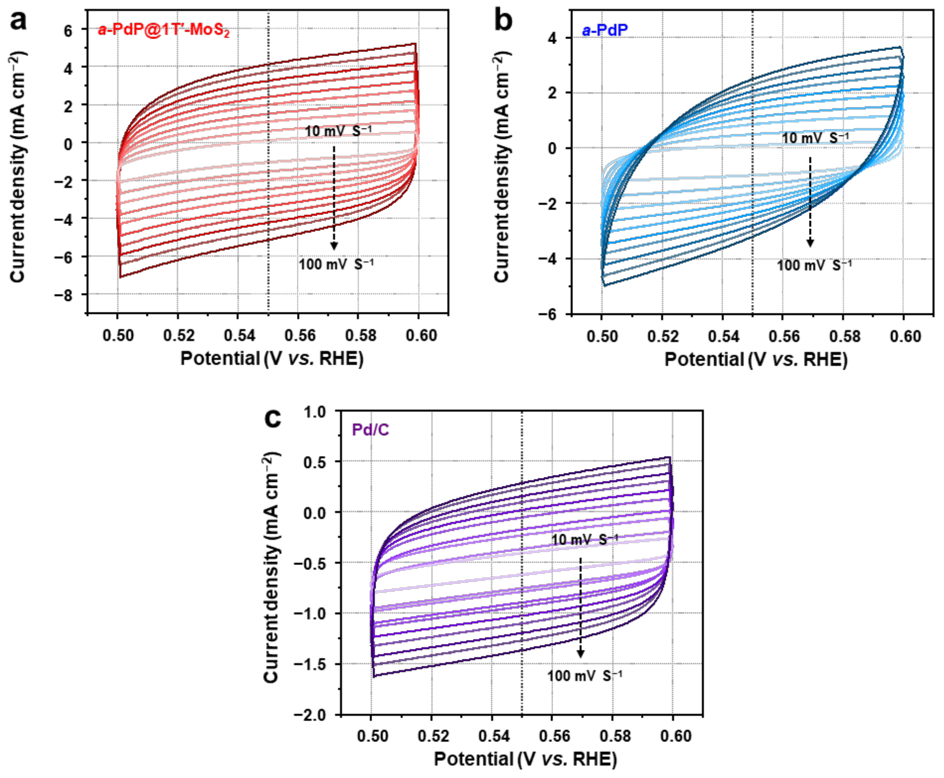

Figure S21. C_{dl} measurements of *a*-PdP@1T′-MoS_2 and other catalysts. (a-c) CV measurements in the potential region of 0.90−1.00 V (*vs.* RHE) for the *a*-PdP@1T′-MoS_2 (a), *a*-PdP (b), and commercial Pd/C (c) at various scan rates from 10 to 100 mV S^{-1}.

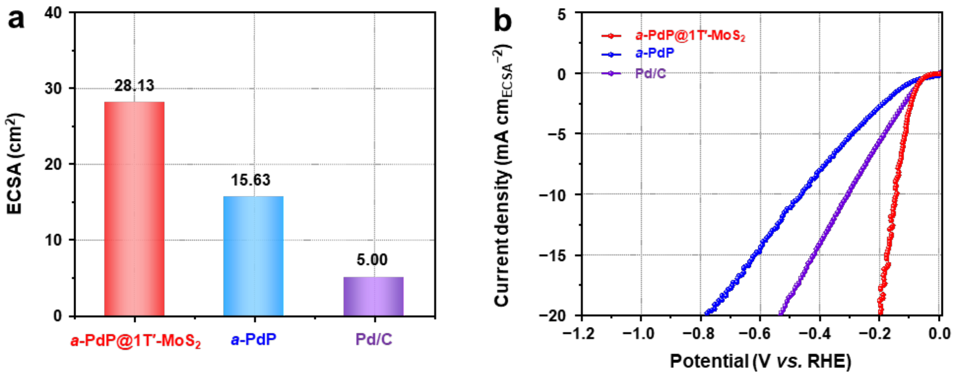

Figure S22. HER activity normalized by ECSA of *a*-PdP@1T′-MoS_2 and other catalysts. (a) The calculated ECSA and (b) the LSV curves normalized by ECSA of the *a*-PdP@1T′-MoS_2, *a*-PdP and commercial Pd/C for HER.

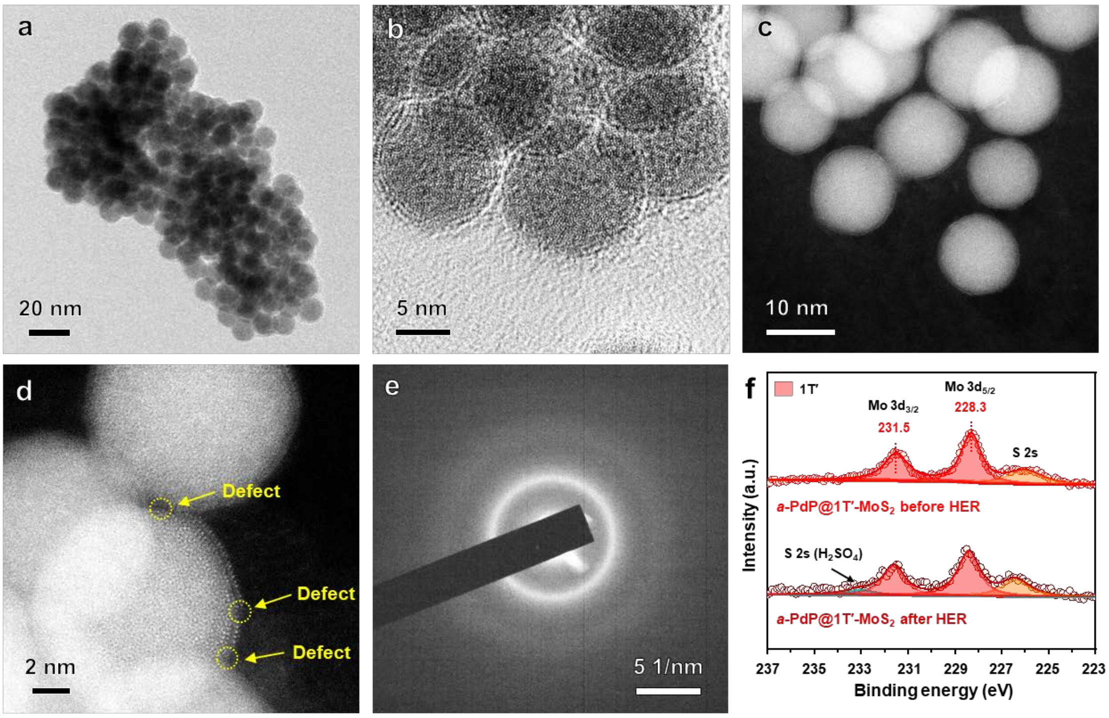

Figure S23. Structural characterization of *a*-PdP@1T′-MoS_2 NPs after HER durability test. (a-c) Low-magnification TEM image (a), HRTEM image (b) and HAADF-STEM image (c) of *a*-PdP@1T′-MoS_2 NPs after HER durability test. (d) Atomic-resolution HAADF-STEM image of the interface between 1T′-MoS_2 MLs and *a*-PdP NPs. The defects in the 1T′-MoS_2 ML after HER durability test are marked with yellow dashed circles. (e) SAED pattern of *a*-PdP@1T′-MoS_2 NPs after HER durability test. (d) High-resolution XPS Mo 3d spectra of *a*-PdP@1T′-MoS_2 NPs before and after HER durability test.

The morphology, crystal phase and electronic structure of *a*-PdP@1T′-MoS_2 NPs after HER durability test have been characterized. As shown in the TEM image (Figure S23a), the spherical morphology of *a*-PdP@1T′-MoS_2 NPs still well maintained. The HRTEM image (Figure S23b) and HAADF-STEM image (Figure S23c) confirm that the *a*-PdP NPs are uniformly encapsulated by the MoS_2 MLs. The atomic-resolution HAADF-STEM image (Figure S23d) reveals that, while maintaining the 1T′ phase, the MoS_2 MLs exhibit a few defects during the long-term durability test, leading to partial exposure of the *a*-PdP surface. The SAED pattern corroborates the amorphous nature of *a*-PdP in the *a*-PdP@1T′-MoS_2 NPs (Figure S23e). The high-resolution XPS Mo 3d spectrum shows no peak shifts after the durability test,

indicating the structure of the 1T′-MoS_2 still maintained (Figure S23f). Note that a small peak in Mo 3d, located at ~233 cm^{-1} (pointed out by the black arrow in Figure S23f), can be attributed to the remaining electrolyte (0.5 M H_2SO_4) on the catalysts.

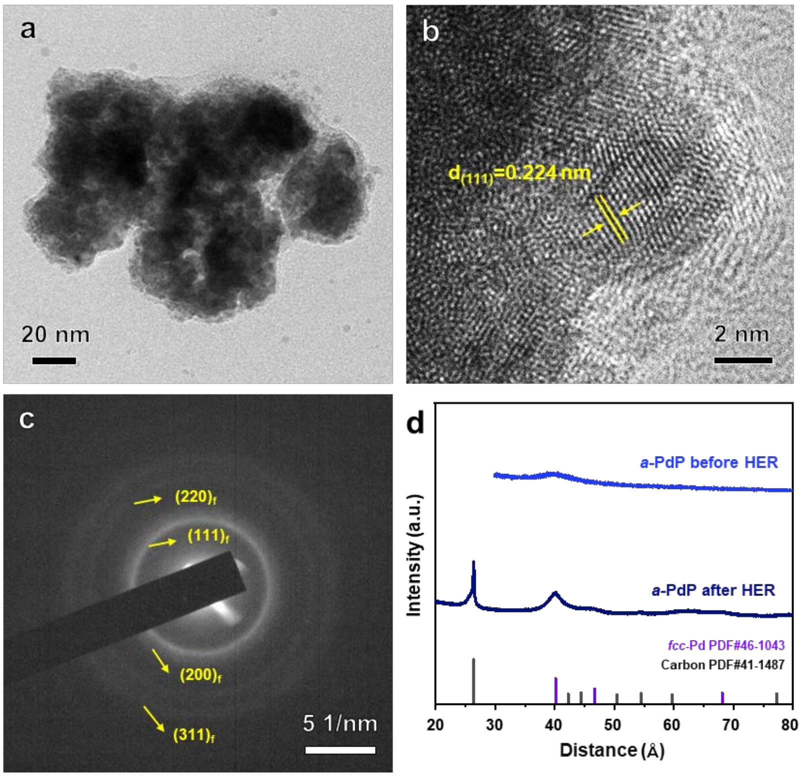

Figure S24. Structural characterization of *a*-PdP NPs after HER durability test. (a-c) Low-magnification TEM image (a), HRTEM image (b), and SAED pattern (c) of *a*-PdP NPs after HER durability test. (d) XRD pattern of *a*-PdP NPs before and after HER durability test.

The morphology, crystal phase and electronic structure of *a*-PdP NPs after HER durability test have been characterized. As shown in the TEM image (Figure S24a), the morphology of *a*-PdP@1T′-MoS_2 NPs has been damaged. Furthermore, HRTEM (Figure S24b) and SAED (Figure S24c) analyses confirm the crystallization of the *a*-PdP NPs. The HRTEM image reveals distinct lattice fringes with a spacing of 0.224 nm, corresponding to the (111) plane of face-centered cubic (*fcc*)-Pd, and the SAED pattern displays polycrystalline rings characteristic of the *fcc* phase. This amorphous-to-crystalline transition is further corroborated by the XRD patterns (Figure S24d), exhibiting sharp diffraction peaks assigned to crystalline *fcc*-Pd, which is different from the *a*-PdP NPs (Figure S1f). Note that the peaks from the carbon substrate are also present in the XRD pattern, since the sample was prepared *via* sonication from the carbon paper.

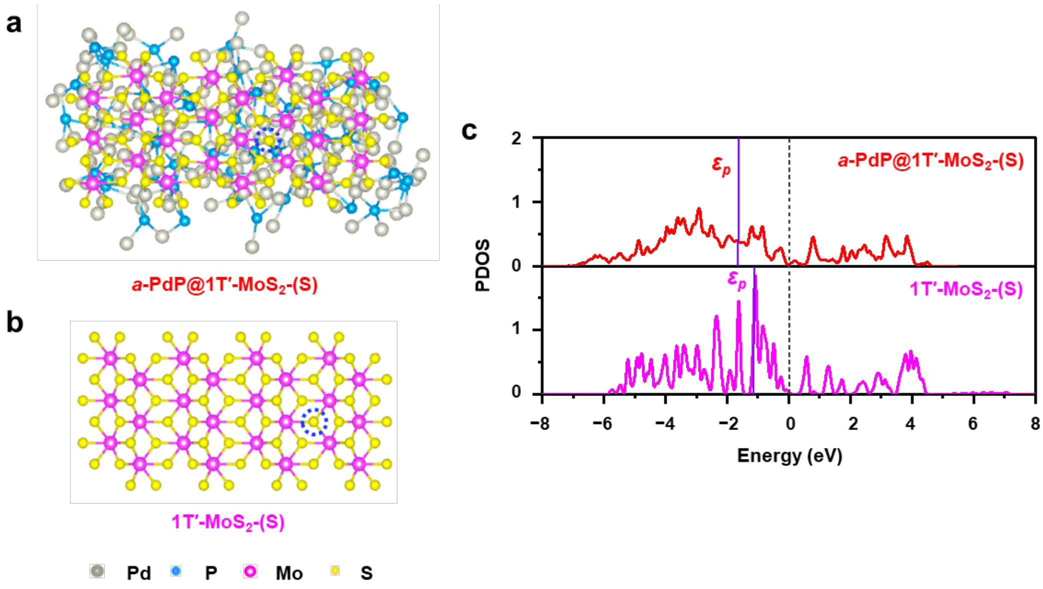

Figure S25. Projected density of states (PDOS) calculations. (a,b) DFT models of S atoms on *a*-PdP@1T′-MoS_2 (a) and 1T′-MoS_2 (b) used for the PDOS calculation. The calculated S atoms are marked with blue dashed circles. (c) PDOS of the *p*-band of S atoms with the corresponding configurations shown in (a,b). The ε_p values are shown in (c). The black dotted line in (c) indicates the Fermi level. The grey, blue, pink and golden balls represent the Pd, P, Mo and S atoms, respectively.

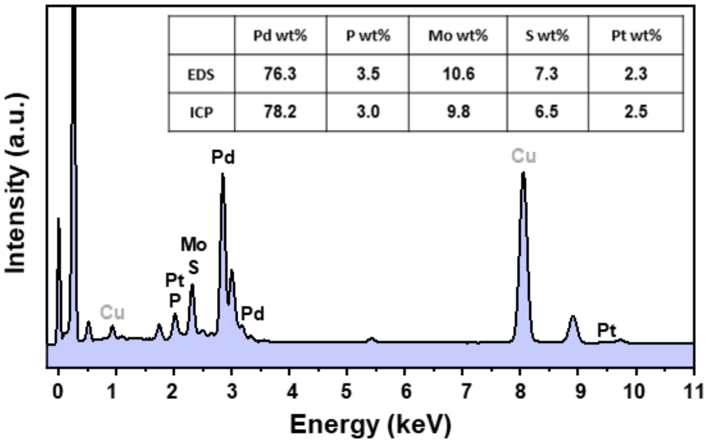

	Pd wt%	P wt%	Mo wt%	S wt%	Pt wt%
EDS	76.3	3.5	10.6	7.3	2.3
ICP	78.2	3.0	9.8	6.5	2.5

Figure S26. STEM-EDS spectrum of *s*-Pt/*a*-PdP@1T′-MoS_2 NPs. The additional Cu signal arises from the Cu grid. Inset: weight ratios of Pd/P/Mo/S/Pt obtained based on EDS and ICP-OES, showing a good agreement.

Table S5. EXAFS fitting parameters at the Pt L_3 edge for various samples*.

Sample	Shell	CN^a	R(Å)^b	σ^2(Å^2)^c	ΔE_0(eV)^d	R factor
fcc-Pt foil	Pt−Pt	12.0	2.76	0.005	7.8	0.002
PtS_2	Pt−S	6.0	2.29	0.007	5.5	0.007
s-Pt/*a*-PdP@1T′-MoS_2	Pt−S	4.0	2.32	0.006	9.2	0.012

^{a}*CN*, coordination number; ^{b}*R*, the distance to the neighboring atom; $^c\sigma^2$, the mean square relative displacement (MSRD); $^d\Delta E_0$, inner potential correction; *R* factor indicates the goodness of the fit. *This value was fixed during EXAFS fitting, based on the known structure of Pt. Fitting range: $3.0 \leq k$ (/Å) ≤ 12.5 and $1.0 \leq R$ (Å) ≤ 3.0 (*fcc*-Pt foil), $3.0 \leq k$ (/Å) ≤ 12.5 and $1.0 \leq R$ (Å) ≤ 2.4 (PtS_2), $3.0 \leq k$ (/Å) ≤ 12.8 and $1.0 \leq R$ (Å) ≤ 2.3(*s*-Pt/*a*-PdP@1T′-MoS_2).

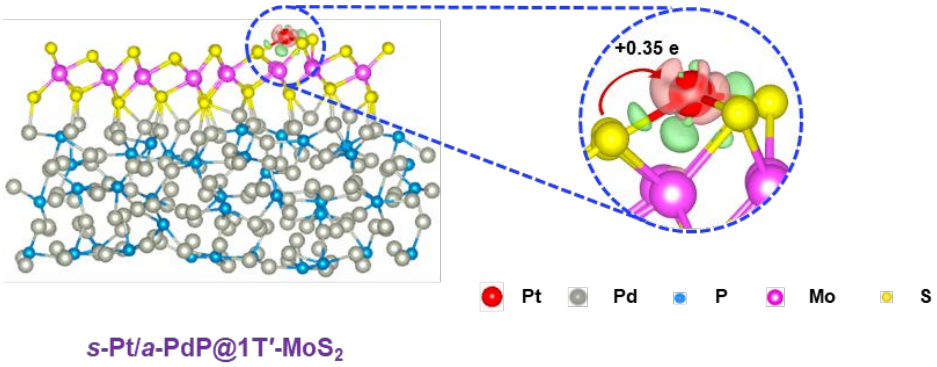

Figure S27. Side views of charge-density difference of *s*-Pt on *a*-PdP@1T′-MoS_2. The green and red colors indicate the electron depletion and accumulation zones, respectively. The red curved arrows indicate the charge transfer from S to *s*-Pt. The red, grey, blue, pink and golden balls represent Pt, Pd, P, Mo and S atoms, respectively.

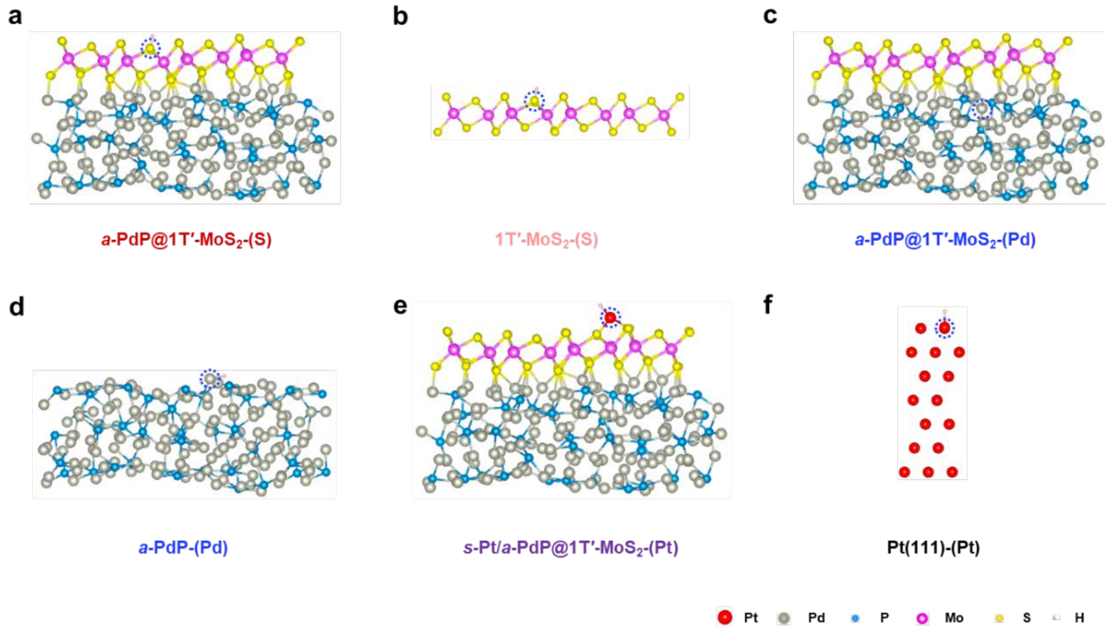

Figure S28. Adsorption configurations of *H intermediates of HER. (a-f) The calculated models of *H adsorbed on S atom in *a*-PdP@1T′-MoS_2 (a), S atom in 1T′-MoS_2 (b), Pd atom in *a*-PdP@1T′-MoS_2 (c), Pd atom in *a*-PdP (d), Pt atom in *s*-Pt/*a*-PdP@1T′-MoS_2 (e), and Pt atom in Pt(111) (f). The calculated metal sites with adsorbed H atoms are marked with blue dashed circles. The red, grey, blue, pink, golden and white balls represent the Pt, Pd, P, Mo, S and H atoms, respectively.

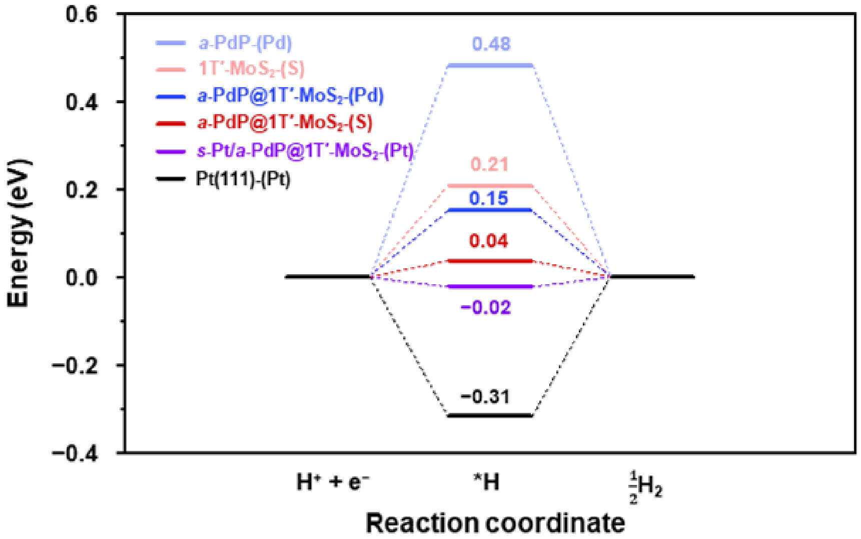

Figure S29. Calculated ΔG_H diagrams for HER.

Table S6. Summary of some representative single-atomically dispersed Pt catalysts for acidic HER reported in the literature and this work.

Materials	Electrolytes	Overpotential [η_{10}, mV]	Tafel slope [mV dec^{-1}]	Long-term stability	Reference
***s*-Pt/*a*-PdP@1T′-MoS$_2$**	**0.5 M H_2SO_4**	**−11.9**	**21.7**	**>100 h@500 mA cm^{-2}**	**This work**
Pt SASs/AG	0.5 M H_2SO_4	−12	29.3	24 h@10 mA cm^{-2}	56
C@Pt-NiFe SAA	0.5 M H_2SO_4	−15	8.9	35 h@10 mA cm^{-2}	57
Pt/TiO$_2$-O$_V$	0.5 M H_2SO_4	−18	12	20 h@10 mA cm^{-2}	58
Pt-GT-1	0.1 M HClO4	−18	24	10000 cycles	59
s-Pt/1T′-MoS$_2$	0.5 M H_2SO_4	−19	118	240 h@1,500 mA cm^{-2}	60
Pt/N-C	0.5 M H_2SO_4	−19	14.2	20 h@10 mA cm^{-2}	61
$Pt_1^{O1}/Ti_{1-x}O_2$	0.5 M H_2SO_4	−20.6	31	520 h@−25 mV	62
Pd/Cu-Pt NRs	0.5 M H_2SO_4	−22.8	25	15 h@−24 mV	63
$Mo_2TiC_2T_x$-Pt$_{SA}$	0.5 M H_2SO_4	−30	30	1000 cycles	64
Pt/2H-WS$_2$	0.5 M H_2SO_4	−32	28	1000 cycles	65
Pt/MXene	0.5 M H_2SO_4	−34	29.7	10000 s@10 mA cm^{-2}	66
Pt$_1$/OLC	0.5 M H_2SO_4	−38	35	100 h@−40 mV	67
Pt-SAs-3D-TaS$_2$	0.5 M H_2SO_4	−39	33.8	6000 cycles	68
Pt$_1$/NMHCS	0.5 M H_2SO_4	−41	56	1000 cycles	69
Pt-AC/DG-150	0.5 M H_2SO_4	−41	37.8	5000 cycles	70
Pt/np-Co$_{0.85}$Se	0.5 M H_2SO_4	−55	35	40 h@−50 mV	71
Pt-SAs/CoNC	0.5 M H_2SO_4	−57	64.5	N.A.	72
Pt1@Fe-N-C	0.5 M H_2SO_4	−60	42	5 h@5 mA cm^{-2}	73
Pt-GDY2	0.5 M H_2SO_4	−75	46.6	10000 s@−95 mV	74
Pt@PCM	0.5 M H_2SO_4	−105	73.6	5 h@−150 mV	75

Abbreviations: SAS, single atomic sites; AG, aniline-stacked graphene; SAA, single-atom alloy; O_V, oxygen vacancies; GT, graphitic tube; N-C, nitrogen-carbon; NR, nanorod; SA, single atom; OLC, onion-like nanosphere of carbon; NMHCS, N-doped mesoporous hollow carbon sphere; AC, atomic cluster; DG, defective graphene; np, nanoporous; CoNC, Co/N co-doped carbon; GDY, graphdiyne; PCM, porous carbon matrix.

Reference

(1) Zhang, Y.; Fang, J.; Zhang, L.; Wei, D.; Zhu, W.; Zhuang, Z. Amorphous palladium-based alloy nanoparticles as highly active electrocatalysts for ethanol oxidation. *Chem. Commun.* **2022**, *58*, 4488–4491.

(2) Li, Z.; Zhai, L.; Zhang, Q.; Zhai, W.; Li, P.; Chen, B.; Chen, C.; Yao, Y.; Ge, Y.; Yang, H.; Qiao, P.; Kang, J.; Shi, Z.; Zhang, A.; Wang, H.; Liang, J.; Liu, J.; Guan, Z.; Liao, L.; Neacșu, V. A.; Ma, C.; Chen, Y.; Zhu, Y.; Lee, C.-S.; Ma, L.; Du, Y.; Gu, L.; Li, J.-F.; Tian, Z.-Q.; Ding, F.; Zhang, H. 1T′-transition metal dichalcogenide monolayers stabilized on 4H-Au nanowires for ultrasensitive SERS detection. *Nat. Mater.* **2024**, *23*, 1355–1362.

(3) Zhou, K. L.; Wang, Z.; Han, C. B.; Ke, X.; Wang, C.; Jin, Y.; Zhang, Q.; Liu, J.; Wang, H.; Yan, H. Platinum single-atom catalyst coupled with transition metal/metal oxide heterostructure for accelerating alkaline hydrogen evolution reaction. *Nat. Commun.* **2021**, *12*, 3783.

(4) Kresse, G.; Furthmüller, J. Efficient iterative schemes for ab initio total-energy calculations using a plane-wave basis set. *Phys. Rev. B* **1996**, *54*, 11169–11186.

(5) Kresse, G.; Furthmüller, J. Efficiency of ab-initio total energy calculations for metals and semiconductors using a plane-wave basis set. *Comput. Mater. Sci.* **1996**, *6*, 15–50.

(6) Perdew, J. P.; Burke, K.; Ernzerhof, M. Generalized gradient approximation made simple. *Phys. Rev. Lett.* **1996**, *77*, 3865–3868.

(7) Grimme, S. Semiempirical GGA-type density functional constructed with a long-range dispersion correction. *J. Comput. Chem.* **2006**, *27*, 1787–1799.

(8) Blöchl, P. E. Projector augmented-wave method. *Phys. Rev. B* **1994**, *50*, 17953–17979.

(9) Monkhorst, H. J.; Pack, J. D. Special points for Brillouin-zone integrations. *Phys. Rev. B* **1976**, *13*, 5188–5192.

(10) Maintz, S.; Deringer, V. L.; Tchougréeff, A. L.; Dronskowski, R. LOBSTER: A tool to extract chemical bonding from plane-wave based DFT. *J. Comput. Chem.* **2016**, *37*, 1030–1035.

(11) Greeley, J.; Jaramillo, T. F.; Bonde, J.; Chorkendorff, I.; Nørskov, J. K. Computational high-throughput screening of electrocatalytic materials for hydrogen evolution. *Nat. Mater.* **2006**, *5*, 909-913.

(12) Yu, Z.; Chen, Y.; Xia, J.; Yao, Q.; Hu, Z.; Huang, W.-H.; Pao, C.-W.; Hu, W.; Meng, X.-M.; Yang, L.-M.; Huang, X. Amorphization activated multimetallic Pd alloys for boosting oxygen reduction catalysis. *Nano Lett.* **2024**, *24*, 1205–1213.

(13) Lai, Z.; He, Q.; Tran, T. H.; Repaka, D. V. M.; Zhou, D.-D.; Sun, Y.; Xi, S.; Li, Y.; Chaturvedi, A.; Tan, C.; Chen, B.; Nam, G.-H.; Li, B.; Ling, C.; Zhai, W.; Shi, Z.; Hu, D.; Sharma, V.; Hu, Z.; Chen, Y.; Zhang, Z.; Yu, Y.; Renshaw Wang, X.; Ramanujan, R. V.; Ma, Y.; Hippalgaonkar, K.; Zhang, H. Metastable 1T′-phase group VIB transition metal dichalcogenide crystals. *Nat. Mater.* **2021**, *20*, 1113–1120.

(14) Lai, Z.; Yao, Y.; Li, S.; Ma, L.; Zhang, Q.; Ge, Y.; Zhai, W.; Chi, B.; Chen, B.; Li, L.; Wang, L.; Zheng, Z.; Gu, L.; Du, Y.; Zhang, H. Salt-assisted 2H-to-1T′ phase transformation of transition metal dichalcogenides. *Adv. Mater.* **2022**, *34*, 2201194.

(15) Liu, L.; Wu, J.; Wu, L.; Ye, M.; Liu, X.; Wang, Q.; Hou, S.; Lu, P.; Sun, L.; Zheng, J.; Xing, L.; Gu, L.; Jiang, X.; Xie, L.; Jiao, L. Phase-selective synthesis of 1T′ MoS_2 monolayers and heterophase bilayers. *Nat. Mater.* **2018**, *17*, 1108–1114.

(16) Yu, Y.; Nam, G.-H.; He, Q.; Wu, X.-J.; Zhang, K.; Yang, Z.; Chen, J.; Ma, Q.; Zhao, M.; Liu,

Z.; Ran, F.-R.; Wang, X.; Li, H.; Huang, X.; Li, B.; Xiong, Q.; Zhang, Q.; Liu, Z.; Gu, L.; Du, Y.; Huang, W.; Zhang, H. High phase-purity 1T′-MoS_2- and 1T′-$MoSe_2$-layered crystals. *Nat. Chem.* **2018**, *10*, 638–643.

(17) Liu, Z.; Nie, K.; Qu, X.; Li, X.; Li, B.; Yuan, Y.; Chong, S.; Liu, P.; Li, Y.; Yin, Z.; Huang, W. General bottom-up colloidal synthesis of nano-monolayer transition-metal dichalcogenides with high 1T′-phase purity. *J. Am. Chem. Soc.* **2022**, *144*, 4863–4873.

(18) Jin, Q.; Liu, N.; Dai, C.; Xu, R.; Wu, B.; Yu, G.; Chen, B.; Du, Y. H_2-directing strategy on in situ synthesis of Co-MoS_2 with highly expanded interlayer for elegant HER activity and its mechanism. *Adv. Energy Mater.* **2020**, *10*, 2000291.

(19) Shi, Y.; Ma, Z.-R.; Xiao, Y.-Y.; Yin, Y.-C.; Huang, W.-M.; Huang, Z.-C.; Zheng, Y.-Z.; Mu, F.-Y.; Huang, R.; Shi, G.-Y.; Sun, Y.-Y.; Xia, X.-H.; Chen, W. Electronic metal–support interaction modulates single-atom platinum catalysis for hydrogen evolution reaction. *Nat. Commun.* **2021**, *12*, 3021.

(20) Gao, M.-R.; Liang, J.-X.; Zheng, Y.-R.; Xu, Y.-F.; Jiang, J.; Gao, Q.; Li, J.; Yu, S.-H. An efficient molybdenum disulfide/cobalt diselenide hybrid catalyst for electrochemical hydrogen generation. *Nat. Commun.* **2015**, *6*, 5982.

(21) Zhang, J.; Wu, J.; Guo, H.; Chen, W.; Yuan, J.; Martinez, U.; Gupta, G.; Mohite, A.; Ajayan, P. M.; Lou, J. Unveiling active sites for the hydrogen evolution reaction on monolayer MoS_2. *Adv. Mater.* **2017**, *29*, 1701955.

(22) Luo, Z.; Ouyang, Y.; Zhang, H.; Xiao, M.; Ge, J.; Jiang, Z.; Wang, J.; Tang, D.; Cao, X.; Liu, C.; Xing, W. Chemically activating MoS_2 via spontaneous atomic palladium interfacial doping towards efficient hydrogen evolution. *Nat. Commun.* **2018**, *9*, 2120.

(23) Padmajan Sasikala, S.; Singh, Y.; Bing, L.; Yun, T.; Koo, S. H.; Jung, Y.; Kim, S. O. Longitudinal unzipping of 2D transition metal dichalcogenides. *Nat. Commun.* **2020**, *11*, 5032.

(24) Deng, S.; Luo, M.; Ai, C.; Zhang, Y.; Liu, B.; Huang, L.; Jiang, Z.; Zhang, Q.; Gu, L.; Lin, S.; Wang, X.; Yu, L.; Wen, J.; Wang, J.; Pan, G.; Xia, X.; Tu, J. Synergistic doping and intercalation: realizing deep phase modulation on MoS_2 arrays for high-efficiency hydrogen evolution reaction. *Angew. Chem. Int. Ed.* **2019**, *58*, 16289–16296.

(25) Wang, T.; Liu, L.; Zhu, Z.; Papakonstantinou, P.; Hu, J.; Liu, H.; Li, M. Enhanced electrocatalytic activity for hydrogen evolution reaction from self-assembled monodispersed molybdenum sulfide nanoparticles on an Au electrode. *Energy Environ. Sci.* **2013**, *6*, 625–633.

(26) Hu, J.; Huang, B.; Zhang, C.; Wang, Z.; An, Y.; Zhou, D.; Lin, H.; Leung, M. K. H.; Yang, S. Engineering stepped edge surface structures of MoS_2 sheet stacks to accelerate the hydrogen evolution reaction. *Energy Environ. Sci.* **2017**, *10*, 593–603.

(27) Su, M.; Zhou, W.; Liu, L.; Chen, M.; Jiang, Z.; Luo, X.; Yang, Y.; Yu, T.; Lei, W.; Yuan, C. Micro eddy current facilitated by screwed MoS_2 structure for enhanced hydrogen evolution reaction. *Adv. Funct. Mater.* **2022**, *32*, 2111067.

(28) Wang, H.; Xiao, X.; Liu, S.; Chiang, C.-L.; Kuai, X.; Peng, C.-K.; Lin, Y.-C.; Meng, X.; Zhao, J.; Choi, J.; Lin, Y.-G.; Lee, J.-M.; Gao, L. Structural and electronic optimization of MoS_2 edges for hydrogen evolution. *J. Am. Chem. Soc.* **2019**, *141*, 18578–18584.

(29) Xie, J.; Zhang, H.; Li, S.; Wang, R.; Sun, X.; Zhou, M.; Zhou, J.; Lou, X. W.; Xie, Y. Defect-rich MoS_2 ultrathin nanosheets with additional active edge sites for enhanced electrocatalytic hydrogen evolution. *Adv. Mater.* **2013**, *25*, 5807–5813.

(30) Shi, Y.; Wang, J.; Wang, C.; Zhai, T.-T.; Bao, W.-J.; Xu, J.-J.; Xia, X.-H.; Chen, H.-Y. Hot

electron of Au nanorods activates the electrocatalysis of hydrogen evolution on MoS_2 nanosheets. *J. Am. Chem. Soc.* **2015**, *137*, 7365–7370.

(31) Pető, J.; Ollár, T.; Vancsó, P.; Popov, Z. I.; Magda, G. Z.; Dobrik, G.; Hwang, C.; Sorokin, P. B.; Tapasztó, L. Spontaneous doping of the basal plane of MoS_2 single layers through oxygen substitution under ambient conditions. *Nat. Chem.* **2018**, *10*, 1246–1251.

(32) Shi, Y.; Zhou, Y.; Yang, D.-R.; Xu, W.-X.; Wang, C.; Wang, F.-B.; Xu, J.-J.; Xia, X.-H.; Chen, H.-Y. Energy Level Engineering of MoS_2 by transition-metal doping for accelerating hydrogen evolution reaction. *J. Am. Chem. Soc.* **2017**, *139*, 15479–15485.

(33) Wang, X.; Zhang, Y.; Si, H.; Zhang, Q.; Wu, J.; Gao, L.; Wei, X.; Sun, Y.; Liao, Q.; Zhang, Z.; Ammarah, K.; Gu, L.; Kang, Z.; Zhang, Y. Single-atom vacancy defect to trigger high-efficiency hydrogen evolution of MoS_2. *J. Am. Chem. Soc.* **2020**, *142*, 4298–4308.

(34) Zheng, Z.; Yu, L.; Gao, M.; Chen, X.; Zhou, W.; Ma, C.; Wu, L.; Zhu, J.; Meng, X.; Hu, J.; Tu, Y.; Wu, S.; Mao, J.; Tian, Z.; Deng, D. Boosting hydrogen evolution on MoS_2 via co-confining selenium in surface and cobalt in inner layer. *Nat. Commun.* **2020**, *11*, 3315.

(35) Duan, H.; Wang, C.; Li, G.; Tan, H.; Hu, W.; Cai, L.; Liu, W.; Li, N.; Ji, Q.; Wang, Y.; Lu, Y.; Yan, W.; Hu, F.; Zhang, W.; Sun, Z.; Qi, Z.; Song, L.; Wei, S. Single-atom-layer catalysis in a MoS_2 monolayer activated by long-range ferromagnetism for the hydrogen evolution reaction: beyond single-atom catalysis. *Angew. Chem. Int. Ed.* **2021**, *60*, 7251–7258.

(36) Li, B.; Nie, K.; Zhang, Y.; Yi, L.; Yuan, Y.; Chong, S.; Liu, Z.; Huang, W. Engineering single-layer hollow structure of transition metal dichalcogenides with high 1T-phase purity for hydrogen evolution reaction. *Adv. Mater.* **2023**, *35*, 2303285.

(37) Lau, T. H. M.; Wu, S.; Kato, R.; Wu, T.-S.; Kulhavý, J.; Mo, J.; Zheng, J.; Foord, J. S.; Soo, Y.-L.; Suenaga, K.; Darby, M. T.; Tsang, S. C. E. Engineering monolayer 1T-MoS_2 into a bifunctional electrocatalyst via sonochemical doping of isolated transition metal atoms. *ACS Catal.* **2019**, *9*, 7527–7534.

(38) Yi, L.; Nie, K.; Li, B.; Zhang, Y.; Hu, C.; Hao, X.; Wang, Z.; Qu, X.; Liu, Z.; Huang, W. Tailoring copper single-atoms-stabilized metastable transition-metal-dichalcogenides for sustainable hydrogen production. *Angew. Chem. Int. Ed.* **2025**, *64*, e202414701.

(39) Li, Y.; Wang, H.; Xie, L.; Liang, Y.; Hong, G.; Dai, H. MoS_2 nanoparticles grown on graphene: an advanced catalyst for the hydrogen evolution reaction. *J. Am. Chem. Soc.* **2011**, *133*, 7296–7299.

(40) Yin, Y.; Han, J.; Zhang, Y.; Zhang, X.; Xu, P.; Yuan, Q.; Samad, L.; Wang, X.; Wang, Y.; Zhang, Z.; Zhang, P.; Cao, X.; Song, B.; Jin, S. Contributions of phase, sulfur vacancies, and edges to the hydrogen evolution reaction catalytic activity of porous molybdenum disulfide nanosheets. *J. Am. Chem. Soc.* **2016**, *138*, 7965–7972.

(41) Yu, X.-Y.; Feng, Y.; Jeon, Y.; Guan, B.; Lou, X. W.; Paik, U. Formation of Ni–Co–MoS_2 nanoboxes with enhanced electrocatalytic activity for hydrogen evolution. *Adv. Mater.* **2016**, *28*, 9006–9011.

(42) Guo, X.; Song, E.; Zhao, W.; Xu, S.; Zhao, W.; Lei, Y.; Fang, Y.; Liu, J.; Huang, F. Charge self-regulation in 1T'''-MoS_2 structure with rich S vacancies for enhanced hydrogen evolution activity. *Nat. Commun.* **2022**, *13*, 5954.

(43) Zhang, H.; Yu, L.; Chen, T.; Zhou, W.; Lou, X. W. Surface modulation of hierarchical MoS_2 nanosheets by Ni single atoms for enhanced electrocatalytic hydrogen evolution. *Adv. Funct. Mater.* **2018**, *28*, 1807086.

(44) Xu, J.; Shao, G.; Tang, X.; Lv, F.; Xiang, H.; Jing, C.; Liu, S.; Dai, S.; Li, Y.; Luo, J.; Zhou, Z.

Frenkel-defected monolayer MoS_2 catalysts for efficient hydrogen evolution. *Nat. Commun.* **2022**, *13*, 2193.
(45) Zhang, J.; Liu, S.; Liang, H.; Dong, R.; Feng, X. Hierarchical transition-metal dichalcogenide nanosheets for enhanced electrocatalytic hydrogen evolution. *Adv. Mater.* **2015**, *27*, 7426–7431.
(46) Luo, Y.; Zhang, S.; Pan, H.; Xiao, S.; Guo, Z.; Tang, L.; Khan, U.; Ding, B.-F.; Li, M.; Cai, Z.; Zhao, Y.; Lv, W.; Feng, Q.; Zou, X.; Lin, J.; Cheng, H.-M.; Liu, B. Unsaturated single atoms on monolayer transition metal dichalcogenides for ultrafast hydrogen evolution. *ACS Nano* **2020**, *14*, 767–776.
(47) Li, H.; Tsai, C.; Koh, A. L.; Cai, L.; Contryman, A. W.; Fragapane, A. H.; Zhao, J.; Han, H. S.; Manoharan, H. C.; Abild-Pedersen, F.; Nørskov, J. K.; Zheng, X. Activating and optimizing MoS_2 basal planes for hydrogen evolution through the formation of strained sulphur vacancies. *Nat. Mater.* **2016**, *15*, 48–53.
(48) Geng, X.; Sun, W.; Wu, W.; Chen, B.; Al-Hilo, A.; Benamara, M.; Zhu, H.; Watanabe, F.; Cui, J.; Chen, T.-p. Pure and stable metallic phase molybdenum disulfide nanosheets for hydrogen evolution reaction. *Nat. Commun.* **2016**, *7*, 10672.
(49) Younan, S. M.; Li, Z.; Yan, X.; He, D.; Hu, W.; Demetrashvili, N.; Trulson, G.; Washington, A.; Xiao, X.; Pan, X.; Huang, J.; Gu, J. Zinc single atom confinement effects on catalysis in 1T-phase molybdenum disulfide. *ACS Nano* **2023**, *17*, 1414–1426.
(50) Lukowski, M. A.; Daniel, A. S.; Meng, F.; Forticaux, A.; Li, L.; Jin, S. Enhanced hydrogen evolution catalysis from chemically exfoliated metallic MoS_2 nanosheets. *J. Am. Chem. Soc.* **2013**, *135*, 10274–10277.
(51) Wu, W.; Niu, C.; Wei, C.; Jia, Y.; Li, C.; Xu, Q. Activation of MoS_2 basal planes for hydrogen evolution by Zinc. *Angew. Chem. Int. Ed.* **2019**, *58*, 2029–2033.
(52) Chen, J.; Liu, G.; Zhu, Y.-z.; Su, M.; Yin, P.; Wu, X.-j.; Lu, Q.; Tan, C.; Zhao, M.; Liu, Z.; Yang, W.; Li, H.; Nam, G.-H.; Zhang, L.; Chen, Z.; Huang, X.; Radjenovic, P. M.; Huang, W.; Tian, Z.-q.; Li, J.-f.; Zhang, H. Ag@MoS_2 core–shell heterostructure as SERS platform to reveal the hydrogen Evolution active sites of single-layer MoS_2. *J. Am. Chem. Soc.* **2020**, *142*, 7161–7167.
(53) Zhang, Y.; Yang, T.; Li, J.; Zhang, Q.; Li, B.; Gao, M. Construction of Ru, O Co-doping MoS_2 for hydrogen evolution reaction electrocatalyst and surface-enhanced Raman scattering substrate: high-performance, recyclable, and durability improvement. *Adv. Funct. Mater.* **2023**, *33*, 2210939.
(54) Kibsgaard, J.; Chen, Z.; Reinecke, B. N.; Jaramillo, T. F. Engineering the surface structure of MoS_2 to preferentially expose active edge sites for electrocatalysis. *Nat. Mater.* **2012**, *11*, 963–969.
(55) Xuan, N.; Chen, J.; Shi, J.; Yue, Y.; Zhuang, P.; Ba, K.; Sun, Y.; Shen, J.; Liu, Y.; Ge, B.; Sun, Z. Single-atom electroplating on two dimensional materials. *Chem. Mater.* **2019**, *31*, 429–435.
(56) Ye, S.; Luo, F.; Zhang, Q.; Zhang, P.; Xu, T.; Wang, Q.; He, D.; Guo, L.; Zhang, Y.; He, C.; Ouyang, X.; Gu, M.; Liu, J.; Sun, X. Highly stable single Pt atomic sites anchored on aniline-stacked graphene for hydrogen evolution reaction. *Energy Environ. Sci.* **2019**, *12*, 1000-1007.
(57) Zhu, Y.; Huang, W.-H.; Shi, X.; Liu, S.; Ait Tamerd, M.; Yang, M.; Li, J.; Yeh, M.-H.; Chang, C.-C.; Cheng, H.; Ma, J. Spatial confinement synthesis of platinum group metal single-atom alloy catalysts for acidic hydrogen evolution. *J. Am. Chem. Soc.* **2025**, *147*, 45893-45905.
(58) Wu, Z.; Yang, P.; Li, Q.; Xiao, W.; Li, Z.; Xu, G.; Liu, F.; Jia, B.; Ma, T.; Feng, S.; Wang, L. Microwave synthesis of Pt clusters on black TiO_2 with abundant oxygen vacancies for efficient acidic electrocatalytic hydrogen evolution. *Angew. Chem. Int. Ed.* **2023**, *62*, e202300406.
(59) Tiwari, J. N.; Sultan, S.; Myung, C. W.; Yoon, T.; Li, N.; Ha, M.; Harzandi, A. M.; Park, H. J.; Kim,

D. Y.; Chandrasekaran, S. S.; Lee, W. G.; Vij, V.; Kang, H.; Shin, T. J.; Shin, H. S.; Lee, G.; Lee, Z.; Kim, K. S. Multicomponent electrocatalyst with ultralow Pt loading and high hydrogen evolution activity. *Nat. Energy* **2018**, *3*, 773-782.

(60) Shi, Z.; Zhang, X.; Lin, X.; Liu, G.; Ling, C.; Xi, S.; Chen, B.; Ge, Y.; Tan, C.; Lai, Z.; Huang, Z.; Ruan, X.; Zhai, L.; Li, L.; Li, Z.; Wang, X.; Nam, G.-H.; Liu, J.; He, Q.; Guan, Z.; Wang, J.; Lee, C.-S.; Kucernak, A. R. J.; Zhang, H. Phase-dependent growth of Pt on MoS_2 for highly efficient H_2 evolution. *Nature* **2023**, *621*, 300-305.

(61) Fang, S.; Zhu, X.; Liu, X.; Gu, J.; Liu, W.; Wang, D.; Zhang, W.; Lin, Y.; Lu, J.; Wei, S.; Li, Y.; Yao, T. Uncovering near-free platinum single-atom dynamics during electrochemical hydrogen evolution reaction. *Nat. Commun.* **2020**, *11*, 1029.

(62) Lu, F.; Yi, D.; Liu, S.; Zhan, F.; Zhou, B.; Gu, L.; Golberg, D.; Wang, X.; Yao, J. Engineering platinum–oxygen dual catalytic sites via charge transfer towards highly efficient hydrogen evolution. *Angew. Chem. Int. Ed.* **2020**, *59*, 17712-17718.

(63) Chao, T.; Luo, X.; Chen, W.; Jiang, B.; Ge, J.; Lin, Y.; Wu, G.; Wang, X.; Hu, Y.; Zhuang, Z.; Wu, Y.; Hong, X.; Li, Y. atomically dispersed copper–platinum dual sites alloyed with palladium nanorings catalyze the hydrogen evolution reaction. *Angew. Chem. Int. Ed.* **2017**, *56*, 16047-16051.

(64) Zhang, J.; Zhao, Y.; Guo, X.; Chen, C.; Dong, C.-L.; Liu, R.-S.; Han, C.-P.; Li, Y.; Gogotsi, Y.; Wang, G. Single platinum atoms immobilized on an MXene as an efficient catalyst for the hydrogen evolution reaction. *Nat. Catal.* **2018**, *1*, 985-992.

(65) Shi, Y.; Ma, Z.-R.; Xiao, Y.-Y.; Yin, Y.-C.; Huang, W.-M.; Huang, Z.-C.; Zheng, Y.-Z.; Mu, F.-Y.; Huang, R.; Shi, G.-Y.; Sun, Y.-Y.; Xia, X.-H.; Chen, W. Electronic metal–support interaction modulates single-atom platinum catalysis for hydrogen evolution reaction. *Nat. Commun.* **2021**, *12*, 3021.

(66) Wu, Y.; Wei, W.; Yu, R.; Xia, L.; Hong, X.; Zhu, J.; Li, J.; Lv, L.; Chen, W.; Zhao, Y.; Zhou, L.; Mai, L. Anchoring sub-nanometer Pt clusters on crumpled paper-like MXene enables high hydrogen evolution mass activity. *Adv. Funct. Mater.* **2022**, *32*, 2110910.

(67) Liu, D.; Li, X.; Chen, S.; Yan, H.; Wang, C.; Wu, C.; Haleem, Y. A.; Duan, S.; Lu, J.; Ge, B.; Ajayan, P. M.; Luo, Y.; Jiang, J.; Song, L. Atomically dispersed platinum supported on curved carbon supports for efficient electrocatalytic hydrogen evolution. *Nat. Energy* **2019**, *4*, 512-518.

(68) Peng, Y.; Yang, X.; Huan, Y.; Wang, J.; Zhou, T.; Lin, H.; Cao, J.; Ding, H.; Fu, J.; Cheng, Y.; Fan, X.; Xia, J.; Zhang, Y. Mutual-activation between doped Pt-single-atoms and basal-plane sites in 1T-TaS_2 nanosheets networks for highly efficient hydrogen evolution. *ACS Nano* **2025**, *19*, 36626-36635.

(69) Kuang, P.; Wang, Y.; Zhu, B.; Xia, F.; Tung, C.-W.; Wu, J.; Chen, H. M.; Yu, J. Pt single atoms supported on N-doped mesoporous hollow carbon spheres with enhanced electrocatalytic H_2-evolution activity. *Adv. Mater.* **2021**, *33*, 2008599.

(70) Cheng, Q.; Hu, C.; Wang, G.; Zou, Z.; Yang, H.; Dai, L. Carbon-defect-driven electroless deposition of Pt atomic clusters for highly efficient hydrogen evolution. *J. Am. Chem. Soc.* **2020**, *142*, 5594-5601.

(71) Jiang, K.; Liu, B.; Luo, M.; Ning, S.; Peng, M.; Zhao, Y.; Lu, Y.-R.; Chan, T.-S.; de Groot, F. M. F.; Tan, Y. Single platinum atoms embedded in nanoporous cobalt selenide as electrocatalyst for accelerating hydrogen evolution reaction. *Nat. Commun.* **2019**, *10*, 1743.

(72) Zhao, Y.; Kumar, P. V.; Tan, X.; Lu, X.; Zhu, X.; Jiang, J.; Pan, J.; Xi, S.; Yang, H. Y.; Ma, Z.; Wan, T.; Chu, D.; Jiang, W.; Smith, S. C.; Amal, R.; Han, Z.; Lu, X. Modulating Pt-O-Pt atomic clusters with isolated cobalt atoms for enhanced hydrogen evolution catalysis. *Nat. Commun.* **2022**, *13*, 2430.

(73) Zeng, X.; Shui, J.; Liu, X.; Liu, Q.; Li, Y.; Shang, J.; Zheng, L.; Yu, R. Single-atom to single-atom grafting of Pt_1 onto Fe−N_4 center: Pt_1@Fe−N−C multifunctional electrocatalyst with significantly

enhanced properties. *Adv. Energy Mater.* **2018**, *8*, 1701345.

(74) Yin, X.-P.; Wang, H.-J.; Tang, S.-F.; Lu, X.-L.; Shu, M.; Si, R.; Lu, T.-B. Engineering the coordination environment of single-atom platinum anchored on graphdiyne for optimizing electrocatalytic hydrogen evolution. *Angew. Chem. Int. Ed.* **2018**, *57*, 9382-9386.

(75) Zhang, H.; An, P.; Zhou, W.; Guan, B. Y.; Zhang, P.; Dong, J.; Lou, X. W. Dynamic traction of lattice-confined platinum atoms into mesoporous carbon matrix for hydrogen evolution reaction. *Sci. Adv.* *4*, eaao6657.
